# Spatio-temporal dynamics of *Plasmodium falciparum* transmission within a spatial unit on the Colombian Pacific Coast

**DOI:** 10.1038/s41598-020-60676-1

**Published:** 2020-02-28

**Authors:** Angélica Knudson, Felipe González-Casabianca, Alejandro Feged-Rivadeneira, Maria Fernanda Pedreros, Samanda Aponte, Adriana Olaya, Carlos F. Castillo, Elvira Mancilla, Anderson Piamba-Dorado, Ricardo Sanchez-Pedraza, Myriam Janeth Salazar-Terreros, Naomi Lucchi, Venkatachalam Udhayakumar, Chris Jacob, Alena Pance, Manuela Carrasquilla, Giovanni Apráez, Jairo Andrés Angel, Julian C. Rayner, Vladimir Corredor

**Affiliations:** 1 Departamento de Microbiología, Facultad de Medicina, Universidad Nacional de Colombia, Bogotá, Colombia; 2 Departamento de Salud Pública, Facultad de Medicina, Universidad Nacional de Colombia, Bogotá, Colombia; 3 Secretaría Departamental de Salud del Cauca, Popayán, Colombia; 4 Departamento de Psiquiatria, Facultad de Medicina, Universidad Nacional de Colombia, Bogotá, Colombia; 5 Post-doctoral fellow, Centro de Hematologia e Hemoterapia (HEMOCENTRO), Universidade Estadual de Campinas (UNICAMP), Campinas, Brazil; 6 Malaria Branch, Division of Parasitic Diseases and Malaria, Centers for Global Health, Centers for Disease Control and Prevention, Atlanta, 30030 GA United States of America; 7 Malaria Programme, Wellcome Sanger Institute, Wellcome Genome Campus, Cambridge, CB10 1SA United Kingdom; 8 Cambridge Institute for Medical Research, University of Cambridge, Cambridge, CB2 0XY United Kingdom; 9 Departamento de Matemáticas, Facultad de Ciencias, Universidad de Los Andes, Bogotá, Colombia; 10 Gestión y desarrollo urbanos, Facultad de Ciencia Política, Universidad del Rosario, Bogotá, Colombia; 11 Department of Mathematics and Statistics, Universidad del Norte, Barranquilla, Colombia; 12 Department of Immunology and Infectious Diseases, Harvard T.H. Chan School of Public Health, Boston, Massachusetts 02115 USA

**Keywords:** Network topology, Ecological epidemiology, Haplotypes, Parasite genetics, Epidemiology

## Abstract

As malaria control programmes concentrate their efforts towards malaria elimination a better understanding of malaria transmission patterns at fine spatial resolution units becomes necessary. Defining spatial units that consider transmission heterogeneity, human movement and migration will help to set up achievable malaria elimination milestones and guide the creation of efficient operational administrative control units. Using a combination of genetic and epidemiological data we defined a malaria transmission unit as the area contributing 95% of malaria cases diagnosed at the catchment facility located in the town of Guapi in the South Pacific Coast of Colombia. We provide data showing that *P. falciparum* malaria transmission is heterogeneous in time and space and analysed, using topological data analysis, the spatial connectivity, at the micro epidemiological level, between parasite populations circulating within the unit. To illustrate the necessity to evaluate the efficacy of malaria control measures within the transmission unit in order to increase the efficiency of the malaria control effort, we provide information on the size of the asymptomatic reservoir, the nature of parasite genotypes associated with drug resistance as well as the frequency of the *Pfhrp2/3* deletion associated with false negatives when using Rapid Diagnostic Tests.

## Introduction

Sustained global malaria control and elimination initiatives, driven by local and international funding agencies, regional malaria control programmes, and the World Health Organisation (WHO), have led to a dramatic decrease in malaria mortality and case incidence in the last 15 years. Worldwide, there has been a reduction in case incidence of 18% and reduction in mortality of 48%, with significant impact in Africa^1^. Although the 2019 WHO Malaria Report suggests that progress may be slowing, malaria elimination is still an active target for many countries, with a global goal of eliminating malaria in at least 35 countries and reducing the global malaria burden by 90% by 2030^2^.

In the Americas, although malaria incidence has decreased globally between 2010 and 2017, incidence has increased since 2014, most significantly in Venezuela, but also in Brazil and Nicaragua. At present, the Americas region contributes an estimated number of more than 900,000 cases, of which Brazil, Colombia, Perú and Venezuela contribute more than 90%. Venezuela alone contributes more than 50% of the total cases of malaria and more that 40% of the total *P. falciparum* cases. Colombia, while contributing only close to 4% of the continent’s *P. vivax* cases, contributes nearly 20% of the *P. falciparum* cases, second only to Venezuela. Although cases in Colombia diminished more than 5-fold between 2001 and 2014, the number of cases doubled to more than 80,000 between 2014-2016, as a result of an increase of *P. falciparum* malaria in the western Pacific region, changing for the first time in more than 40 years the *P. vivax*/*P. falciparum* ratio (42%/56%). This increase in the number of malaria cases coincides with a weakening of the surveillance and control systems and the lack of timely availability of antimalarial treatment as a result of the decline of Global Fund assistance^3–6^, as well as an increase in gold mining activities in the region associated with malaria epidemics^7,8^ and the continuous arrival of migrants from Venezuela^9^. This trend is in opposite direction to the goals of the National Malaria Control Programme of eliminating urban malaria transmission and progressively reduce malaria morbidity by 50% in endemic areas between 2012–2021^10^.

A major obstacle for malaria control programmes in ecologically and socially complex endemic areas is to define circumscribed spatial units where feasible malaria control measures can be implemented, allocating resources in the most cost-effective way. These spatial units can initially be defined in terms of the area covered by sites where health providers offer malaria diagnostics and treatment (i.e. health facilities catchment areas), the transmission intensity, the dynamics by which human populations are acquiring the infection or infected individuals are entering the spatial unit, and the effectiveness of the applied control measures. However, in practical terms, there is often little knowledge of the different parameters that define malaria transmission systems on the part of control programmes, making the control effort ineffectual or not optimal from a cost-effectiveness point of view. An example of this is the lack of knowledge of many intrinsic (parasite, mosquito, human) and extrinsic (control and prevention, social, demographic, behavioral, economic, political, environmental) or biotic and abiotic factors defining the malaria burden^11,12^.

To improve the efficacy of control measures it is critical to define malaria spatial units at the micro-epidemiological level, identify spatial heterogeneity of transmission and assess the effectiveness of malaria control programmes within these units. Pioneering studies in other locations have generated fine scale maps of malaria endemicity and identified spatial units that have higher transmission than their surroundings, otherwise known as hotspots^13,14^. The stability of these hotspots^15^ and the relationship between transmission intensity and malaria hotspots are areas of ongoing study^16^. Using different malaria transmission variables at the micro-epidemiological level, maps and models have been produced to guide National Control Programmes in Africa and Southeast Asia^12,17–22^. By contrast in the Americas, where the dynamics of malaria transmission is different from Africa and Southeast Asia due to differences in vector bionomics, social conditions or parasite genetic structure among others, relatively few studies have attempted to identify transmission heterogeneity, whether using epidemiological data^23^, serological tools^24,25^ or assessing the effect of ecological differences on malaria transmission^26^.

In low transmission areas, as in the Americas, *P. falciparum* parasites display low genetic diversity and a high level of monogenomic or clonal infections (i.e. Multiplicities of Infection (MOI) close to 1) resulting in high inbreeding, small effective population sizes, low effective recombination and strong linkage disequilibrium. Moreover, populations in low transmission settings tend to be highly structured over small geographical distances in contrast to parasite populations in high transmission areas. This results in clonal or highly clonal populations persisting over large periods of time, which allows tracking infections in time and space making it possible to infer mobility and migration patterns and determine the genetic composition of parasites during epidemic peaks^27–31^. In regions like the Americas, genetic analysis linked to epidemiological information and spatial mapping of malaria cases could result in a powerful tool to characterize the genetic structure of parasite populations within a spatial malaria transmission unit, infer connectivity patterns in time and space and identify importation and introduction as well as sources and sinks of parasite infections within a spatial transmission unit^31,32^.

**Figure 1:**
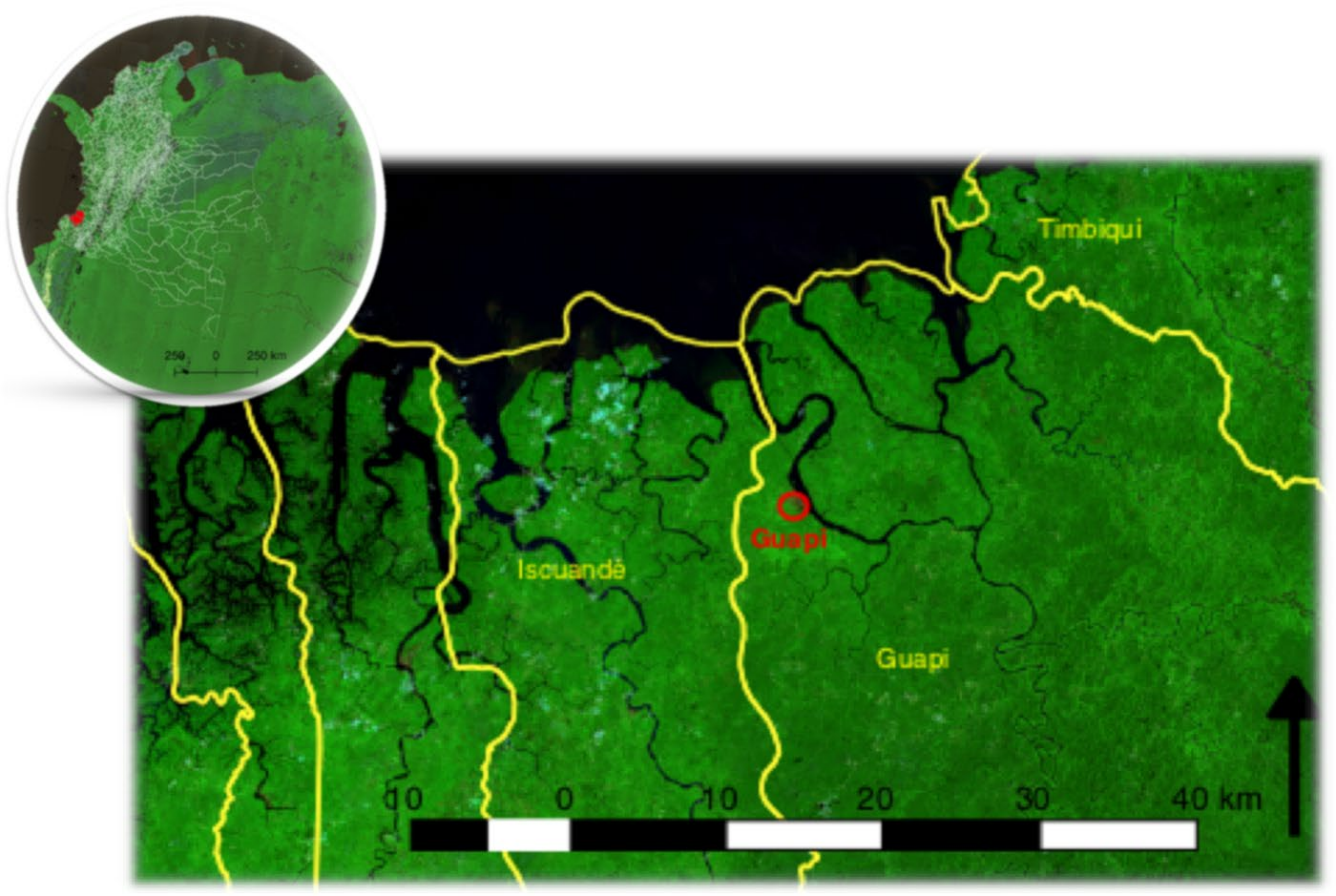
Study site. Malaria diagnostic posts located in the Guapi region in the south Pacific departmental province of Cauca in Colombia. Guapi constitutes the main diagnosis post in the region. Other posts are run by volunteers that do not provide a permanent service. Maps created using Landsat 8 composite images freely available at Google Earth Engine (https://earthengine.google.com/)^77^ and QGIS version 2.18 (http://www.qgis.org)^78^.

The Guapi municipality in the western Pacific coast of Colombia is a 2,688 km${}^{2}$ territory of >30,000 inhabitants, with about half inhabiting the town of Guapi on the banks of the Guapi river (Fig. 1). The town is connected through a network of river and seaways (there are no roads outside the urban area) to a number of hamlets in the region whose populations fluctuate between 60–1,500 inhabitants. Malaria transmission in Guapi is endemic and unstable, and is almost exclusively caused by *P. falciparum* (Fig. S1 1). The town has an established health post that registers cases of malaria, and one of the aims of the Colombian malaria control programme is to eliminate urban malaria transmission in Guapi by 2021^10^. However, it was not clear at the outset of this study whether cases registered in the town of Guapi are the result of urban malaria transmission, or due to cases diagnosed in Guapi but acquired in surrounding hamlets. Likewise, the dynamics of malaria transmission, i.e. the heterogeneity of transmission and the connectivity between the number of human settlements in the area, were poorly understood, as well as many of the determinants of the efficacy of the malaria control programme such as the extent of the asymptomatic reservoir, the nature of drug resistance genotypes circulating in the area or the efficacy of Rapid Diagnostic Tests (RDTs). The malaria control programme relies on microscopic diagnosis and to a lesser extent on RDTs that are used when epidemics occur in sites of difficult access. RDTs used by the Regional Control Programme rely on the detection of the parasite’s Histidine Rich Protein 2 (PfHRP2) whose cognate gene has been shown to be deleted in isolates in the Americas and some regions in Africa and India^33–36^. *P. falciparum* anti-malarial treatment is provided according to guidelines by the Ministry of Health and consists of arthemeter/lumefantrine. However, the uncovering of mutations associated to artemisinin resistance in Guyana^37^, the massive migration flow from Venezuela to Colombia and other South American countries^9^ and the use of non-recommended treatments and self-medication with anti-malarial drugs acquired through the informal sector or the use of anti-malarial drugs at subtherapeutic doses^38^, threatens to thwart the malaria control effort.

To provide malaria control programmes with a tool to deliver interventions aimed at malaria elimination, here we define for the first time a spatial malaria transmission unit for the Guapi area in the Pacific coast of Colombia, using samples collected specifically for this purpose between 2014 and 2017. We characterize the heterogeneity of transmission within this spatial transmission unit, determine the population structure of parasites circulating within it and evaluate connectivity patterns between sites contributing malaria cases in the area based on parasite genetic data and epidemiological information. To illustrate the necessity to consider the efficacy of interventions within the malaria spatial unit and convert this spatial unit into an operational malaria control unit, for which the effectiveness of the malaria control programme can be evaluated, we estimate some important variables: the size of the asymptomatic reservoir, the frequency of *P. falciparum* alleles associated with drug resistance and the prevalence of the *Pfhrp2/3* deletion on which the efficacy of RDTs depend.

## Results

### Guapi spatial malaria transmission unit: origin and temporal distribution of cases

**Figure 2:**
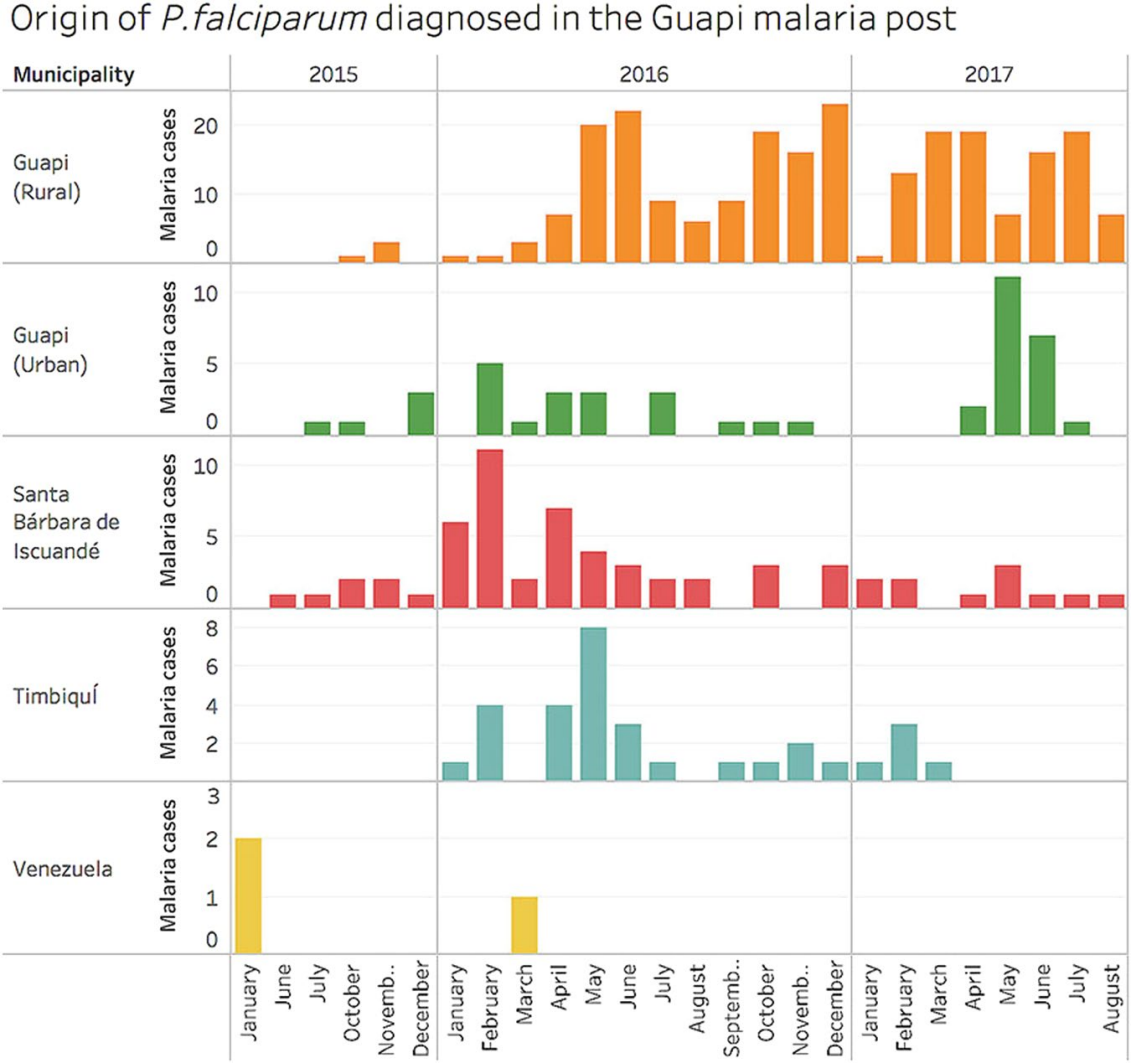
*P. falciparum* infections in the study region according to the municipality or region of origin. More detailed information is provided in Table S1 1.

To generate a high resolution picture of malaria in the Guapi region (Fig. 1), we carried out systematic passive surveillance between November 2014 and August 2017 by collecting samples from cases that register at local clinics. In total, we microscopically diagnosed 497 cases of malaria; 435 (87.5%) at the local health post in Guapi and the remainder (12.4%) at part-time microscopy posts in El Cuerval (7%), Chanzará (2.8%) and Carmelo (2.6%). The majority of symptomatic cases were diagnosed at the Guapi health post. This location serves as a health provider for a wide range of communities within and outside Guapi, so self-reported origins of patients at this post were also recorded (Fig. 2; Table S1 1). Only 18% of the cases diagnosed at the Guapi health post originated in peri-urban Guapi, whereas more than 80% had their origin within rural communities outside urban Guapi. Five of the sites (Guapi, Carmelo, Limones, Quiroga, San José de Guare) each contributed on average 14% of cases each, indicating local foci of transmission, while another 29 sites each contributed less than 0.6% of cases on average (Table S1 1), indicating a broad distribution of transmission around the region. The temporal distribution of cases was not uniform. The four rural localities in the Guapi municipality each experienced epidemic peaks in either 2016 or 2017, whereas urban Guapi experienced cases throughout the study period (Fig. 2; Table S1 1). Excluding urban Guapi, all sites that contributed large numbers of cases were close to sites where gold-mining activities were taking place. As expected 99.6% of cases were due to *P. falciparum*, with only a single case of *P. vivax* detected, as defined by species-specific PCR. Of the 497 cases, 188 (37.8%) were women and 306 (61.6%) were men (three undetermined), suggesting that malaria in this region may in part be an occupationally acquired disease, as men tend to work in regions where transmission is higher. The average age of those infected was 25.4, while the age range was 1–82. The average parasitaemia estimated by microscopy was 8,469 parasites/$\mu l$ (range 48–148,000 parasites/$\mu l$) and 219 gametocytes/$\mu l$ (range 32–592 gametocytes/$\mu l$), which compared to previous studies in Colombia, represents relatively high gametocytemia^39,40^. More than 96% of malaria cases diagnosed at the Guapi malaria health facility originated within a 25 km radius of the town of Guapi. We therefore define the Guapi malaria transmission unit as the area where the catchment facility captures 95% of cases (Fig. S1 4).

### *Plasmodium falciparum* population structure

We used both microsatellites and SNP data to establish the genetic structure of the *P. falciparum* populations diagnosed in Guapi, Chanzará, Carmelo and Cuerval using Bayesian inference STRUCTURE software (Materials and Methods). STRUCTURE uses a probabilistic Bayesian model-based method to assign individuals to clusters or populations based on the allele frequencies or genotypes at each locus. The method assumes a model of K number of populations, and individuals are probabilistically assigned to a given population represented by a colour. Admixed individuals will show the proportion of the genotype belonging to a given population. Since mutation rates for microsatellites and individual point mutations differ extensively^41–43^, affecting their potential power of population discrimination^44^, we tested the hypothesis of various parasite populations circulating in the area using both a panel of 6 neutral microsatellites genotyped on 148 individual samples and a number of SNP barcode datasets from samples diagnosed at the malaria posts in Guapi, and El Cuerval. Initially, several SNP barcode datasets were constructed across a range of data thresholds: excluding loci with more than 10–40% missing sites, and excluding samples with missing data above 5–10%. All datasets predicted the same number of populations, therefore for subsequent analyses we used the more stringent dataset excluding loci with missing sites above 10 percent and above 5 percent for individual samples (Barcode 10–5, 117 samples and 49 loci).

Two different population clusters (K = 2) were detected when using the microsatellite dataset. Both were found at the Guapi health post at nearly equal frequency (Cluster 1: 0.52; Cluster 2: 0.48) but parasites diagnosed in Chanzará and Carmelo were mainly of Cluster 2 type (0.92 and 0.97 respectively). On the contrary, parasites diagnosed in El Cuerval belonged in a higher proportion to the Cluster 1 type. Likewise, parasites from infections originating in different sites in the municipality of Santa Bárbara de Iscuandé (e.g. Chanzará) were mainly of the Cluster 2 type (0.86) while those originating in the municipality of Timbiquí (e.g. El Cuerval) were of the Cluster 1 type (0.68). Parasites from the rural area of Guapi were mainly of the Cluster 2 type (0.88) while those originating in the urban area of Guapi were of the Cluster 1 type (0.72). We computed average distances (expected heterozygosity) between individuals in the same diagnostic site and found that the largest distance between individuals corresponded to Guapi (0.193) and the shortest to Chanzará (0.009) and Carmelo (0.010). This suggests that the town of Guapi is a demographic parasite sink, receiving infections from surrounding municipalities.

**Figure 3:**
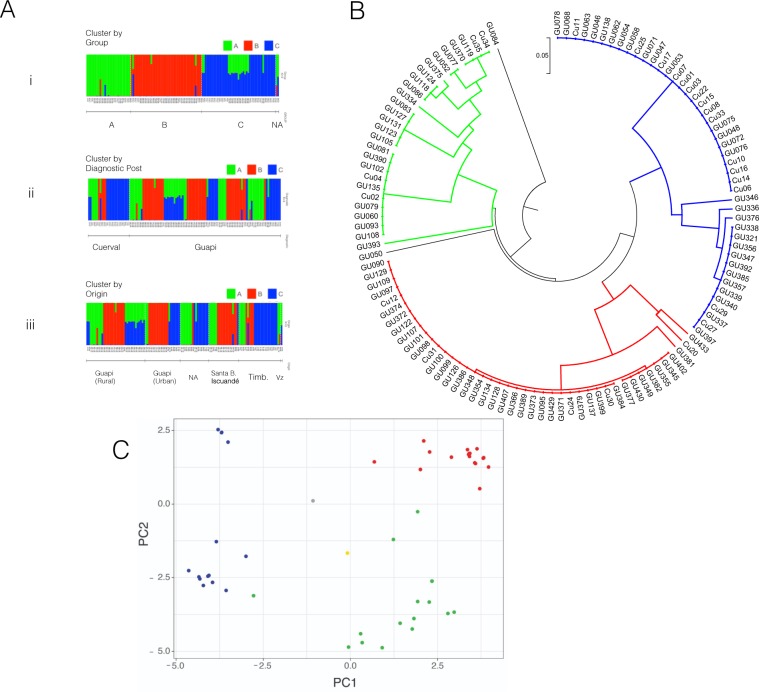
Population structure of *P. falciparum* parasite populations circulating in the Guapi area. (**A**) Populations inferred by STRUCTURE using 117 samples and 49 loci. Identified subpopulations (K = 3) are depicted by colours A (Green), B (Red) and C (Blue). Vertical bars represent each of the isolates (X-axis). Colours represent the fraction (Y-axis) of each isolate with respect to the inferred populations (K = 3). Upper row (i), populations inferred by STRUCTURE (K = 3), Middle row (ii): distribution of populations at the diagnostic sites. Lower row (iii): distribution of populations according to the locality of origin. (**B**) UPGMA dendrogram of 117 samples and 49 SNP positions used in STRUCTURE analysis. Distances were computed using p-distances. Populations inferred are the same as those inferred by STRUCTURE (same colour code) except that GU084 and GU050 were excluded from any group. GU084 is a sample originating at the northern edge of the distribution and GU050 is a sample originating in Venezuela. Sample Cu20 is assigned in STRUCTURE analysis to population C. (**C**) Principal Component Analysis (PCA) of the above dataset. Individual samples are coloured based on the clusters identified by STRUCTURE (green, red, and blue) and the clades identified on the UPGMA dendrogram. Sample originating from Venezuela (GU050) and sample GU084 are depicted in grey and yellow respectively.

Inference of population structure using SNP barcode 10–5 revealed three populations (K = 3) (hereafter referred to as populations A (30 individuals), B (42) and C (45)). All three populations were found at the Guapi (A: 0.29, B: 0.41, C: 0.29) and El Cuerval malaria diagnostic posts (A: 0.68, B: 0.16, C: 0.16), although in different proportions. We looked at the spatial distribution of these three parasite populations between six defined regions according to the reported origin of infections (i.e. urban Guapi, rural Guapi, the municipality of Santa Bárbara de Iscuandé, Nariño and the municipality of Timbiquí, and parasites diagnosed at the malaria post in Guapi that originate in the surrounding area but without a precise reported location) (Figs. 3A and S1 3). Although all parasite populations are present in all regions, the frequency of each population was different at each reported location origin, suggesting some geographic structuring (Guapi urban: A: 0.11, B: 0.50, C: 0.39; Guapi rural: A: 0.23, B: 0.37, C: 0.40; Timbiquí, Cauca: A: 0.21, B: 0.21, C: 0.58; Santa Bárbara de Iscuandé, Nariño: A: 0.28, B: 0.67, C: 0.06) (Fig. S1 3, Fisher’s exact test p-value = 0.002). In particular samples from the municipality of Santa Bárbara de Iscuandé, Nariño are constituted mostly of population B while samples from Cuerval, Timbiquí, have a high representation of population C (Odds ratio = 38.0, p-value = 0.00017). Interestingly, all populations coexisted in urban and rural Guapi, mirroring the results from the microsatellite data and emphasising the role of Guapi as a sink for infections from the surrounding area. In addition, populations B and C are constituted of highly related individuals, i.e. the expected heterozygosity between individuals for populations B and C is 0.025 and 0.084 respectively while that for population A is 0.147. This suggests that populations B and C are mainly clonal populations.

To assess the clonality of the populations we generated a UPGMA tree using genetic distances (p-distances) from the 117 samples barcode dataset at 49 loci (barcode 10–5), (Fig. 3B). As inferred with STRUCTURE the dendrogram reveals three main clusters or subpopulations. Two clusters (B and C) are constituted by highly similar genotypes, mirroring the heterozygosity data noted above, while a third cluster (A) consisted of genotypes that are more distantly related to each other (overall mean distance of the tree: 0.224; within mean distances for groups A, B and C: 0.147, 0.025 and 0.084 respectively). Clusters B and C are therefore constituted by a large number of identical or nearly identical parasites. For example, if we analyse a subgroup of 40 individuals (88.9%) in group B, the mean distance within that group is 0.0025 of which 81% are identical. Similarly, a subgroup of 29 individuals from group C (69.1%) are identical. Clonal populations persisted during all the study period and are distributed all along the spatial unit (Fig. S2 12). Interestingly, a single sample originating from Venezuela and a sample from Playa Chacón, Timbiquí, display a large mean distance against all groups (0.329 and 0.405 respectively). Principal Component Analysis (PCA) also reveals a similar population structure of parasites circulating in the Guapi area (Fig. 3C). Therefore, multiple different methods of analysing the genotype data (barcode 10-5) all suggest that three parasite populations coexist in the area. Although most individual parasite infections were of low complexity (MOI = 1), eight percent were the result of polyclonal infections all of which had an estimated MOI equal to two (RealMcCOIL); these were all excluded from the analysis above.

### Topological data analysis

**Figure 4:**
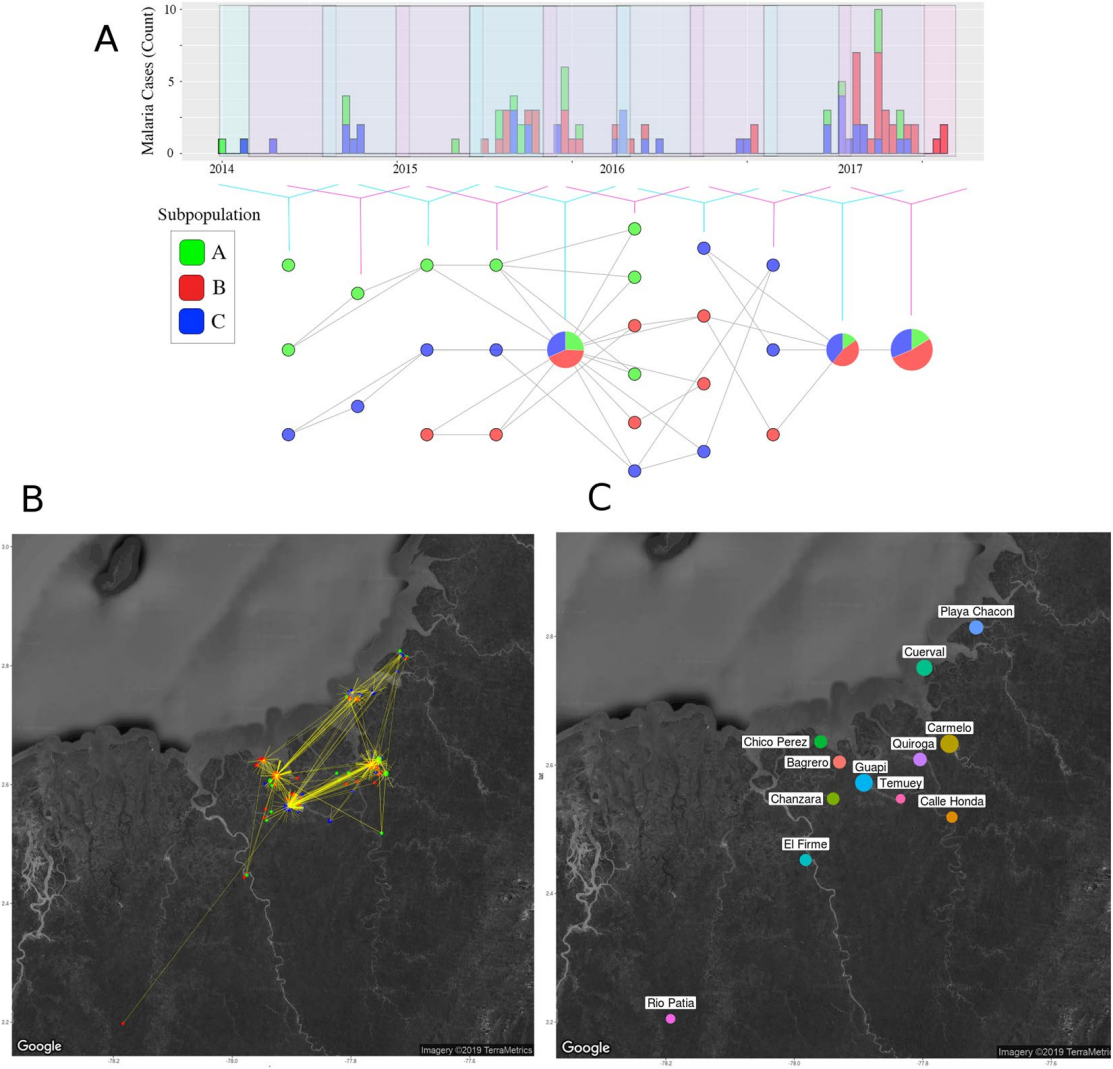
TDA network configuration of the parasite population diagnosed in Guapi between 2014-2017 and Malaria Transmission Unit. (**A**) Cases in the study area are represented using *Mapper* as a tool, using as preassigned filter functions genetic distances (p-distances) and time. The graph is the 1-skeleton of the TDA. Colours represent subpopulations identified through STRUCTURE (A: green; B: red; C: blue). Circle sizes represent the number of genotypes assigned to a given subpopulation over overlapping time periods. Note that the clustering algorithm of the TDA can assign genotypes to two different clusters while STRUCTURE assigns them to the same subpopulation, thus having separate clusters of the same colour over a single interval. (**B**) TDA map of network connections between cases diagnosed in the Guapi area between 2014–2017 and representation of Guapi Malaria Transmission Unit. Nodes are represented by a colour according to the identified parasite subpopulation (A: green; B: red; C: blue). This is the Point Intersection Graph representation of (**A**). Nodes are connected if they appear on different clusters of (**B**) (i.e. if there is an overlapping or “intersection” between clusters). In this graph, nodes point towards cases with the highest in-degree centrality. The size of the nodes correspond to the in-degree of the node (i.e the number of directed edges pointing towards it). (**C**) Geographical locations found after executing hierarchical clustering on the coordinates of the samples. The location of the point is computed using the mean of the coordinates and the size is proportional to the number of its elements. Notice that connectivity of case clusters does not necessarily occur in the geographical region with the highest diversity (Guapi), and that while cases of all genetically defined subpopulations are observed in urban Guapi, the connection between regions surrounding Guapi seems to play a fundamental role in the dissemination of genetic variants during epidemic outbreaks. Maps where created using the R package: *ggmap*^79^.

To investigate the nature of the connectivity between sites within the spatial malaria transmission unit, pairwise genetic distances of the samples used for the STRUCTURE, dendrogram, and PCA analyses, and time of infection, were used as a distance measure and filter function to cluster data through Topological Data Analysis (TDA) (Supplementary Material 2). A network representation of the connectivity of parasite populations was generated (Fig. 4A) and projected onto a spatial map to visualise the spatio-temporal dynamics of parasite infections. This representation allowed us to quantify the spatial centrality of particular infections (i.e. the relative importance of nodes within a network, see Supplement 2) and the connectivity between infections and locations (Figs. S2 10 and S2 11).

The network reveals the genetic relatedness and frequency of circulating parasites and their continuity over time. The genetic composition of circulating parasites during the time of the study shows that subpopulation C was present throughout the study period, while subpopulation B was absent at the beginning. Subpopulation A was not detected at time points in 2016, but reappeared in 2017 as part of a cluster including all subpopulations during epidemic peaks. The data show a pattern of endemic and unstable malaria transmission punctuated by epidemic peaks during which all subpopulations increase in frequency. Cases with the highest Pagerank (i.e. the centrality of a node given the number of linking neighbours and their relative importance within the network) and betweenness centrality (i.e. the importance of a node in linking paths between other nodes) values were observed during epidemic intervals (2015 and 2017), while cases with high betweenness centrality but low Pagerank values were observed during inter-epidemic intervals (2016) (Figs. 4A and S2 10). Both of these groups of cases highlighted by the TDA analysis belong to populations B and C.

A spatial representation of the network shows the dynamics of infection within the Guapi transmission Unit (Fig. 4B). A number of clusters with high centrality (Pagerank values for some nodes of the geographic network: Guapi 0.40, Carmelo 0.26, Bagrero 0.10) appear at sites that can be considered as relevant in the transmission of malaria in the area. These sites or nodes are located northeast of the town of Guapi as indicated by yellow arrows (Fig. 4B) and are located in close proximity to a site of gold mining activities (Fig. 4B,C; Quiroga, Carmelo). This indicates an area of higher than average transmission and where the probability of recombination may increase (83.3% of multiple infections occurred in 2017 and 72.2% in those places undergoing epidemic events).

### Asymptomatic malaria

**Figure 5:**
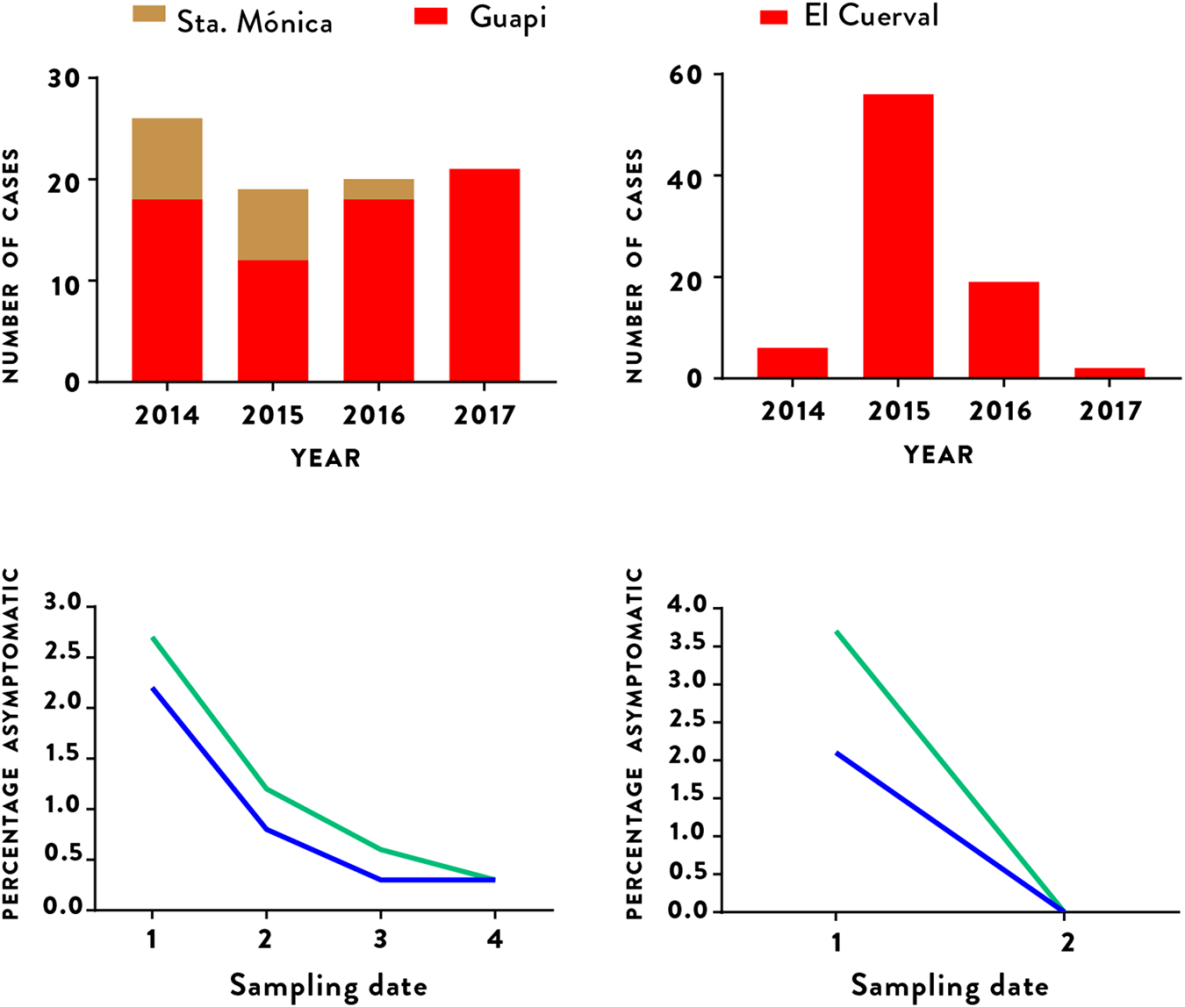
The relationship between asymptomatic individuals and malaria incidence in Guapi (left column) and El Cuerval (right column). Asymptomatic individuals found to have parasitemia during the study period by microscopy (blue line) or PCR (green line) at different time points (lower row) and the number of cases diagnosed during routine surveillance in the town of Guapi (red bars) and in the Santa Mónica neighborhood of Guapi (mustard bars) and El Cuerval (red bars, upper row). Asymptomatic individuals in Guapi were sampled in four occasions in the Santa Mónica neighbourhood (1: April (2015), 2: October (2015), 3: April (2016), 4: October (2016) (see Methods)) and are represented as a fraction (brown) of the total cases (red) in the town of Guapi. Asymptomatic prevalence by PCR in Guapi were: 1: April-2015 [6/226 (2.7%); CI 95%: 0.9-5.8], 2: October (2015) [3/250 (1.2%); CI 95%: 0.2–3.5], 3: April (2016) [2/298 (0.6%); CI 95%: 0.1-2.4], 4: October (2016) [1/303 (0.3%); CI 95%: 0.1–1.9]. Asymptomatic prevalence by microscopy in Guapi were: 1: April (2015) [5/226 (2.2%); CI: 0.01–5.1], 2: October (2015) [2/250 (0.8%); CI 95%: 0.2–2.9], 3: April (2016) [1/298 (0.3%); CI 95%: 0.1–1.9], 4: October (2016) [1/303 (0.3%); CI 95%: 0.1–1.9]. In El Cuerval two sampling events were performed at time points with different malaria incidence (1: December, 2015, 2: May, 2017). As the number of cases diminishes so does the number of asymptomatic individuals with parasitemia. Asymptomatic prevalence by PCR in El Cuerval were: 1: December, 2015 [7/188 (3.7%); CI 95%: 1.5–7.7], 2: May, 2017 [0/103 (0%); CI 95%: 0–1.2]. Asymptomatic prevalence by microscopy in EL Cuerval were: December, 2015 [4/188 (2.1%); CI 95%: 0.8–5.3], 2: May, 2017 [0/103 (0%); CI 95%: 0–1.2]. CI = Confidence Interval.

In addition to the passive detection of clinical cases at health posts, during the study period four community surveys were also carried out in Santa Mónica in urban Guapi to identify cases of asymptomatic malaria. As described in the Methods, a random selection of an average of 270 individuals were included in each survey, with malaria diagnosed by thick smear microscopy and dried blood spots stored for subsequent molecular diagnosis by PCR. Similar surveys were also carried out twice in the rural hamlet of El Cuerval, once in December 2015 a few months after an epidemic peak, and once in May 2017 during a period where few cases were registered. All inhabitants present in El Cuerval at the time of each visit were sampled. 1,368 blood samples in total were studied across the six surveys (1,077 in Santa Mónica and 291 in El Cuerval). Of these, 688 (50.3%) were from women, 680 (49.7%) were from men and their average age was 28 (range 2–96). Thirteen (0.9%) of the 1,368 samples from asymptomatic individuals tested positive for malaria parasites by thick slide microscopy, while PCR amplification of the 18S rRNA gene identified 19 (1.5%) as positive for *P. falciparum* infection. Of the 13 cases diagnosed by microscopy, 11 were confirmed by PCR, while 8 cases were detected by PCR only and hence were sub-microscopic infections. All infections were due to *P. falciparum* in both microscopy and PCR diagnosis. The average parasitaemia was 1,419 parasites/$\mu l$ (range 48–5,944 parasites/$\mu l$) and 71.7 gametocytes/$\mu l$ (range 32–121 gametocytes/$\mu l$). The precise figures as well as socio-demographic information of individuals surveyed are shown in Table S1 2. The number of asymptomatic individuals with detectable parasitemia decreased as the number of diagnosed cases in Santa Mónica decreased. Likewise, the number of asymptomatics in El Cuerval was highest after an epidemic peak and decreased to zero after seventeen months when no malaria cases were detected (Fig. 5).

### Genotyping *Pfhrp2* and *Pfhrp3* deletions and drug resistance loci

31 randomly selected parasite samples from symptomatic individuals reporting at the Guapi Malaria Post were tested for deletion of *Pfhrp2* and *Pfhrp3* genes, whose products are components of the most commonly used rapid diagnostic tests. Two samples (6.2%) displayed a deletion at the *Pfhrp2* gene but not the flanking regions. Remarkably, 27 samples (87.1%) exhibited a deletion of the *Pfhrp3* gene. Several samples also presented, in addition, a deletion of the neighbouring genes, 8 showing a deletion of the 3'and 5' flanking regions of PF3D7 1372100 and PF3D7 1372400, 17 of the PF3D7 1372100 region only, 1 of the PF3D7 1372400 region only, and two samples (6.2%) displayed a deletion of both the *Pfhrp2* and *Pfhrp3* genes. This indicates that at least for the *Pfhrp3* gene several independent events have resulted in a *Pfhrp3* deletion (Table S1 3).

Genotypes associated to resistance to the drugs chloroquine, sulfadoxine/pyrimethamine, and artemisinin were assessed by PCR and Sanger sequencing (Materials and Methods). The majority of samples displayed genotypes associated with resistance to chloroquine, pyrimethamine and sulfadoxine (approx. 80%) (mutant positions underlined) (Fig. S1 2). Most *Pfcrt* haplotypes were of the CVMNT (4/87) and CVMET (79/87) type previously reported in the Pacific coast of Colombia. *Pfdhfr* haplotypes are distributed as follows: CNCSI (18/87; 20.7%), CICSI (1/87; 1.2%), CNCNI (64/87; 73.6%), CICN I (3/87; 3.5%) and RICN I (2/87; 2.3%). The majority of the observed *Pfdhps* haplotypes correspond to the wild type SAKAA (15/74; 20.3%) and the single mutant SGKAA (52/74; 70.3%). All samples (n = 230) sequenced for the propeller domain of the *Kelch13* locus were of the wild type, providing no evidence for artemisinin resistance associated genotypes. Several of the resistance genotypes were identified for the first time in the western Pacific region of Colombia: *Pfcrt* SVMNT (2/87; 2.3%), *Pfdhfr *RICNI (2/91; 2.2%) and *Pfdhps* SGEGA (1/72; 1.4%). All were identified in samples diagnosed at the Guapi Malaria post but originating from a gold-mining area in Venezuela, aside from *Pfdhps* FGKAA (2/74; 2.7%) which was of uncertain origin (Fig. S1 2).

## Discussion

Malaria elimination requires the identification of clearly defined spatial malaria transmission units where the control effort can by directed in cost-effective ways and where progressive, feasible goals towards elimination can be established. This requires an understanding of the dynamics of malaria transmission and the barriers hindering elimination goals within the spatial unit. Here, we initially defined a spatial malaria transmission unit by carrying out passive case collections and taking travel histories at local health posts. These revealed that infections diagnosed at the Public Malaria Facility in the town of Guapi, in the western Pacific region of Colombia, originated mostly (>95%) in 33 small rural settlements and in peri-urban neighborhoods of the town of Guapi. The area covered by the catchment facility is an area of 25 km radius, having the town of Guapi as its centre. Cases originating outside this spatial unit represented less than 5% of the cases and they originate from as far as Venezuela (Fig. 4). This has important implications for the malaria control programme: 1) the spatial size covered by the catchment facility is small enough to implement focussed control measures aimed at the elimination of malaria transmission in the area and 2) it reveals that urban malaria transmission is much lower than predicted by the current reporting system, suggesting that if appropriate malaria control measures are provided, urban malaria transmission can be eliminated within a reasonable timeframe.

A characterization of malaria transmission within this spatial unit showed that malaria transmission is heterogeneous in time and space. Three transmission patterns were observed: peri-urban transmission occurring close to mosquito breeding sites and representing a relatively small proportion of the total number of cases (less than 20% of overall cases); low endemic transmission (24.5% of total cases) occurring mainly in small settlements (60–200 inhabitants) in the rural areas of the municipalities of Santa Bárbara de Iscuandé, Guapi and Timbiquí, and epidemic transmission (56.7% of total cases), occurring in rural settlements (Limones, Carmelo, Quiroga, San José de Guare) of relatively high human population density (densely populated human settlements of approx 1,000 inhabitants), connected through riverways and located in close proximity to sites where gold mining activities were taking place at the time of the study. This coincided with a weakening of the malaria microscopy network in the Pacific region, the arrival of migrants from Venezuela to Colombia, the increase of malaria cases in the Pacific region associated to *P. falciparum* infections and an increase of mainly illegal-gold mining activities in large areas of Colombia.

Diagnostic SNP combinations defining parasite subpopulations can be used to infer sources, routes of importation and mobility patterns that can constitute a useful tool when difficulties arise in using other data to quantify the flow of individuals between locations (e.g. scant data on commercial boat mobility or low mobile phone coverage and use)^31^. We assessed the population structure of *P. falciparum* populations circulating in the area using a SNP barcode corresponding to 49 independent loci (see Methods), taking advantage of the long term persistence of clonal lineages in South American populations of *P. falciparum*^28^ which allows tracking parasite populations in time and space. Long term persistance of clonal lineages is probably due to the low diversity of *P. falciparum* populations in the Americas^27^ as a consequence of the bottleneck resulting from its relatively recent introduction during the slave trade^45,46^, the strong selective pressure by antimalarials^47,48^ and the relatively low mosquito Blood Index Feeding rates in South American anophelines as compared to high transmission areas in Africa^49–53^. This results in a low proportion (8% in this study) of polyclonal infections, low MOIs and highly clonal lineage infections persisting over time^28^.

We identified three *P. falciparum* subpopulations (A, B and C), circulating and coexisting in the area, of which two, B and C, were highly clonal. This may be the result of low effective recombination frequency and past waves of clonal expansion. Previous studies using a set of 250 SNPs have found four populations circulating in Guapi between 1999-2003, of which two lineages constituted 98% of the samples^28^. Using a set of six microsatellite loci we only detected two parasite populations. The higher resolution provided by SNP data may be due to the fact that three of the six microsatellite loci used (TA1, TA109, C2M34) have low diversity in these populations^43,54^. We used the Evanno method to estimate the optimal number of clusters detected by STRUCTURE without making any assumption on the demographic history of parasite populations, i.e. the contribution of selection, drift or past introduction of new genotypes. However, there are limitations to the method: it is sensitive to the sample size and it may not detect sub-structuring of the populations^55^. Here, for example, it cannot discriminate parasites originating from Venezuela from other populations which can otherwise be visualised in the distance-based dendrogram (Fig. 3B) or in PCA analysis (Fig. 3C). Since genetic distance between clusters may be due to divergence, admixture between the observed populations or with unknown "ghost" populations, a more detailed sampling of parasites populations from other regions outside the spatial transmission unit is needed, including a higher density of SNPs that would be required to evaluate demographic and epidemiological, or ancestral population admixture history hypotheses^56^.

The spatial frequency distribution of parasite populations shows that all three populations are observed in the town of Guapi, which therefore constitutes a sink for cases across the rural area of the Guapi municipality. At the extremes of the spatial transmission unit some populations are more abundant (Fig. S1 3) suggesting that parasite populations originate in different geographical areas. Evidence for this has been previously provided, showing the differential distribution of 4 different parasite populations along the Colombian Pacific coast^28^. The fact that all parasite populations are observed in the town of Guapi reveals its nature as a receptor of individuals seeking malaria diagnosis and treatment. With a population of nearly 18,000 inhabitants, it is the major human centre in the municipality and constitutes a hub for commercial activities providing different facilities such as education centres for children and the youth and health services through private and public providers. Private and public malaria diagnostic posts in Guapi report malaria cases to the regional and national health authorities. Critically, these reports assume all cases as being the result of local transmission, an assumption that this work clearly proves is not valid.

In this work we defined imported cases in the town of Guapi as those cases where the individual has been continuously present at a site outside urban Guapi in the previous two weeks before diagnosis. There are two limitations to this definition: first, individuals may have acquired the infection in places close, but not exactly at, to the indicated site of residence (e.g. at nearby mining sites) and second, infections may become patent after acquisition in the previous three to four weeks. Although these caveats mean that precision may be lacking with respect to the exact time and site of infection acquisition, the general conclusion that the majority of cases diagnosed in Guapi are imported and originate in defined areas in the surroundings is supported by their association to mining sites, known foci of infections. The large number of sites outside urban Guapi, suggests that if anything, the analysis underestimates the number of cases classified as rural or peri-urban.

TDA combines genetic and epidemic variables to explore transmission patterns over space and time. As mentioned above, we observe heterogeneous transmission in the Guapi area and surroundings. The town of Guapi is highlighted by all descriptive measures, as is Bagrero (geographic Pagerank, case Pagerank and betweenness) during epidemic and inter-epidemic intervals. The area of El Cuerval at the northen part of the malaria transmission unit is highlighted by high betweenness centrality in both networks. A strong interaction between Guapi and Carmelo is emphasised by the TDA, suggesting that epidemic areas such as Carmelo and Quiroga are connected by their epidemic dynamics. Bagrero and El Cuerval appear as secondary in the map, however, their betweenness centrality suggests they both play an important role in connecting regional parasite populations.

This suggests that El Cuerval and Bagrero possibly constitute semi-independent hubs within the transmission unit, which play a role in the reintroduction of parasites (due to the high betweenness centrality of the cases during non-epidemic years). A third transmission hub in the area comprised by Carmelo and Quiroga is characterised by high Pagerank values in the geographic network, with similarly high values in Pagerank and betweenness centrality in the case network, particularly during epidemic years (2017 mainly).

It is important to note that network descriptive statistics is highly susceptible to the projection used to express the output of TDA onto a map and to the choice of parameters in the TDA algorithm. Here, we propose a method to interpret TDA in the context of genetic and epidemic variables for georeferenced cases. However, other possible projections should be explored in further work, which will also confirm whether the observed patterns of case betweenness and Pagerank centrality and epidemic and inter-epidemic intervals adequately describe the dynamics of the malaria transmission unit. Furthermore, as data become available, studies with larger sample sizes will confirm whether our results are biased by the limited number of sequenced infections.

Interestingly, epidemics were not found to be due to the expansion of a single clonal population^57,58^ but to an increase of all three parasite populations circulating in the area (Fig. 4). This suggests that epidemic peaks are due to an increase in mosquito density due to the opening of breeding sites in mining areas^51,59^, an increase in human population densities at mining sites and the human mobility across the mining area and the populated settlements.

Although the number of cases originating outside the defined 25 km radius transmission unit is small (3%), its importance is revealed by the presence of cases originating in Venezuela. Interviews with patients at the diagnostic post revealed that infected individuals acquired the infection at gold mining areas in Venezuela. Parasites derived from those individuals carried genotypes associated with resistance to chloroquine, pyrimethamine and sulfadoxine that were previously unseen in the Pacific coast of South America^57,60,61^. Data from Colombia’s National Statistics Department show that in 2005, 2.8% of households in Guapi had some type of experience as international migrants, of which 47.2% had Venezuela as destination^62^. Colombian migration to Venezuela was mainly due to economic or humanitarian reasons. However, due to Venezuela’s ongoing crisis more than 20,000 Colombian residents have returned from Venezuela, in particular since 2015. In addition, between 2015 and 2017 more than 550,000 Venezuelan citizens migrated to Colombia, posing an important challenge to the health system and to Colombia’s malaria control effort^9^.

We observe that more than 80% of multiclonal infections (15 out of 18) occurred during epidemic peaks. This suggests that under this transmission pattern, epidemic peaks constitute potential recombination hotspots. If this is the case, we would expect successive waves of clonal expansion followed by recombination events. Since the net effect of recombination between two different clones would be a decrease in the branch length linking the two clones and an increase in the branch length within a particular clonal population, the dendrogram in Fig. 3 suggests this possibility. High density genotyping to infer segmental Identity by Descent (IBD) would be desirable to map recombination events that may prove to be hallmarks of epidemic events.

These results may have important consequences for planning control interventions. For example, Guapi operates as a receptor of infections, maintains some local transmission and constitutes a hub for the dissemination of infections. However, transmission is localised to a few neighbourhoods and vector control may constitute an efficient control measure (e.g. identification and control or elimination of mosquito breeding sites). For sites with high Pagerank centrality, where localised epidemics occur, timely diagnosis and treatment may be required to suppress further transmission while sites with high betweeness but low Pagerank centrality may require continuous surveillance of cases and active case detection. Also, as epidemics occur at sites that are the most populated in the area, close to sites where mining activities take place and in areas that are connected through river or seaways, which allows for the dissemination of infections, microscopy diagnostic posts should be considered as well as a system capable to reach the mining population.

An operational definition of a malaria control unit requires an evaluation of the efficacy of control measures and the factors, intrinsic (biological) and extrinsic (social, political, demographic, etc), hampering the malaria control effort. This would allow determination of the feasibility and cost of gradual and attainable goals towards malaria elimination. As an example of the difficulties encountered by the control system we estimated the size of the asymptomatic reservoir in the neighbourhood of Guapi that has historically contributed most of the malaria cases and in a hamlet representative of many of the locations where endemic malaria transmission occurs. We also determined the nature of the genotypes associated to drug resistance circulating in the area and the frequency of the *Pfhrp2/3* deletion that contributes false negative diagnostic results when using RDTs.

The number of identified asymptomatic individuals was low (<5%) as compared with previous studies in the Colombian Pacific coast (>20%). This may be due to the different sampling methods. Here, we actively searched for asymptomatic individuals from a randomly chosen sample in Santa Mónica, Guapi or from the total population of El Cuerval, Timbiquí, at different time points. Others used a reactive active search strategy whereby asymptomatic individuals were searched for in a given perimeter from an identified symptomatic index case during an epidemic peak^63^. Here we show that the number of asymptomatic individuals decreased as malaria incidence decreased. The epidemiological relevance of asymptomatic individuals may therefore depend on the rate of natural clearance of parasite infections (average persistence of asymptomatics) and the mosquito biting rate.

All parasites displayed genotypes associated to chloroquine resistance and 80% displayed genotypes associated with resistance to pyrimethamine and sufadoxine. This is important in terms of the efficacy of the control measures since, as recently documented, 32% of individuals in a location in the south Pacific coast were found to use chloroquine as auto medication according to Saker-Solomon tests directed at detecting chloroquine in urine^38^. Likewise we have surveyed local pharmacies in Guapi and found that both chloroquine and sulfadoxine-pyrimethamine can be obtained over the counter outside the health providers’ system.

Finally, 6% of parasite samples analysed exhibited a deletion in both the *Pfhpr2 and 3* genes suggesting that at least 6% of diagnostic tests performed with RDTs within the transmission unit are false negatives.

An understanding of malaria transmission at the micro epidemiological level can define a malaria transmission unit by assessing the effective range of the catchment facility, the heterogeneity of transmission and identifying sources and sinks of infection. Such information is necessary to direct the malaria control effort, but in order to build an effective, spatially defined, operational malaria control unit, an assessment of the efficacy of the individual control measures that takes into account entomological, environmental, social, and demographic factors, as well as factors limiting the accessibility to the health system or limiting the establishment of efficient control measures is also required. This work is an important step to defining such a control unit for the Guapi region, and some of the findings can be extrapolated to similar urban areas along the Pacific Coast.

## Materials and Methods

### Ethical oversight

The study followed national and international ethical standards. The project was presented to and discussed by local government health authorities and ethical approval was obtained from the Ethics Committee of the Faculty of Medicine at the Universidad Nacional de Colombia (approval number 127-14). Written informed consent was obtained from all participants both at the health centres and in the surveys, and in the case of children, from their parents or guardians^64–66^.

### Study area

The study was carried out in the Cauca Department on the Pacific Coast in southern Colombia between November 2014 and August 2017 (Fig. 1). The Pacific Coast of the Cauca department is only accessible by river or plane and is an intertropical convergence area in which, due to high temperatures, air masses rise causing heavy rainfall, a phenomenon that is constant almost all year. The climate of the region is warm (average temperature $2{9}^{\circ }$C) and humid (average humidity 87.5%). Passive sample collection was carried out at diagnostic posts in the town of Guapi (${2}^{\circ }33{\prime} 53.75{\prime\prime}$N; $7{7}^{\circ }52{\prime} 52.43{\prime\prime}$W), and small villages in El Carmelo (Guapi municipality) (${2}^{\circ }38{\prime} 10.52{\prime\prime}$N; $7{7}^{\circ }45{\prime} 35.21{\prime\prime}$W), Chanzará, Nariño (Santa Bárbara de Iscuandé municipality) (${2}^{\circ }32{\prime} 51.14{\prime\prime}$N; $7{7}^{\circ }56{\prime} 21.98{\prime\prime}$W) and El Cuerval in the Timbiquí municipality (${2}^{\circ }45{\prime} 1.81{\prime\prime}$N; $7{7}^{\circ }47{\prime} 59.40{\prime\prime}$W). Active sampling was carried out in the neighbourhood of Santa Mónica in the town of Guapi and the village of El Cuerval which is one hour from Guapi by boat. Guapi is located on the banks of the Guapi River, within a system of forests and mangroves, while El Cuerval is an estuarine mangrove system on a beach on the Pacific Ocean. According to the National Administrative Department of Statistics, in 2017 the total population in the Guapi municipality was 29,867, of which 18,277 lived in urban areas and the total population in El Cuerval was approximately 200 individuals; in both places the Unsatisfied Basic Need Index is >85%. In both areas, the principal cause of malaria is *P. falciparum* (more than 95% of the cases), because as noted above 97% of the population is Afro-Colombian with a resulting high prevalence of Duffy negativity. According to the Health Office of Cauca Department, in 2014, 2015, 2016 and 2017 (epidemiological period 10) the annual parasite index (API) in the Cauca department was 1.8, 0.8, 3.8 and 3.3 respectively. The number of malaria cases recorded in each of these years was 400, 128, 483 and 32 in the town of Guapi and 6, 56, 19 and 1 in El Cuerval respectively^67^. During the study period routine malaria control activities were performed by the Cauca Health Office (Secretaria Departamental de Salud del Cauca), i.e. treatment of positive malaria cases, insecticide spraying, control of breeding sites in urban Guapi and uneven distribution of insecticide-treated bednets in locations where cases were reported.

### Sample collection

Individuals self-reporting with malaria associated symptoms (fever, chills and sweating) were diagnosed by microscopy at the continuously operating public local health post in Guapi (N = 435) during the entire study period (2014–2017). Symptomatic cases that had a positive diagnosis provided both blood spots stored on Whatman 3 filter paper and 4 ml of venous blood obtained by venipuncture into ethylenediaminetetraacetic acid (EDTA) vacutainer tubes after providing an informed consent. All individuals who tested positive for malaria infection were treated with 20 mg of artemether and 120 mg of lumefantrine in tablets taken twice daily for three days, regardless of whether they were enrolled in the study. At the time of diagnosis a patient travel survey was also performed, and cases were classified as local if the patient had been continuously present at the same site for the previous two weeks before diagnosis; otherwise they were classified as imported and the location where they had spent the majority of the last two weeks was recorded as the likely site of origin of the case.

### Active malaria case detection: asymptomatic malaria

At both active detection locations, Guapi and El Cuerval, a census was performed at the beginning of the study period. Each inhabitant was registered with a consecutive number and their name, age, identification document and gender were recorded. In addition, the coordinates of each house were recorded using a Global Positioning System (GPS), and all data recorded in a database. Surveys were then carried out four times in Santa Mónica: in April and October 2015 and in April and October 2016. On average 200 individuals were randomly selected and invited to be tested for asymptomatic parasitemia, undergo a physical examination, and take part in a survey of demographic variables, including previous exposure to malaria. Venous blood samples were collected by fingerprick for use in microscopy diagnosis via thick blood smear^68^. Quality control was performed by a second expert evaluator. Asymptomatic individuals who were found to have parasitemia by microscopy on day 0 were followed up clinically on days 7 and 14, and thick blood smears were evaluated on both days. Blood samples were also stored on Whatman 3M filter paper for use in subsequent PCR diagnosis and genotyping. Surveys were carried out twice in El Cuerval: in December 2015 and in May 2017. However, because El Cuerval is significantly smaller, a non-probabilistic selection was made and almost the entire population of 200 individuals was evaluated; otherwise the survey and sample collection process were the same.

Malaria positive individuals over two years old testing negative for malaria by clinical evaluation in the previous week (symptoms of fever, chills and sweating and heart rate, respiratory rate, axillary temperature, blood pressure testing and general physical examination), and who had given informed consent, were included in the study. Individuals who had been diagnosed with malaria in the previous week and individuals who were receiving treatment at the time of evaluation were excluded, along with pregnant women and infants under the age of 2. All individuals who tested positive received the standard treatment for uncomplicated *P. falciparum* malaria in Colombia^69^, which is a fixed combination of 20 mg of artemether and 120 mg of lumefantrine in tablets taken twice daily for three days.

### Molecular diagnosis

Molecular diagnosis was performed using *Plasmodium* species-specific nested PCR on dried blood spots. Haemoglobin was removed from 3 mm diameter blood spots in filter paper by adding 30 µl of Molecular Grade Water (Promega DW0991), and incubating at 50 °C for five minutes, 21 °C for 15 seconds, 50 °C for 1.5 minutes, and 21 °C for 15 seconds. Water was subsequently removed and discarded. *Plasmodium* 18S rRNA was amplified using a modified direct-nested PCR modification approach^55^, using a Phusion Blood Direct PCR Kit (Thermo Fisher Scientific) and species-diagnostic primers described previously^56^. In the first round of amplification, 20 µl of reaction mixture containing 10 µl 2X Pushion blood PCR Buffer (200 µM deoxynucleoside triphosphates [dNTPs], 3 mM of MgCl_2_), 0.3 µl of Phusion blood DNA polymerase (0.6 U/reaction) and 0.25 µM of each primer (rPlu5 and rPlu6) were directly applied on 0.2 ml tubes after haemoglobin removal. An aliquot from this first amplification was then added to the second nested amplification step. For species-specific diagnosis, different PCRs were conducted in parallel. One, that contained primers rfal1-rfal2 and rviv-rviv2 for *P. falciparum* and *P. vivax* respectively, and another containing primers rmal1-rmal2 for *P. malariae*. PCR reactions were performed in 10 µl of reaction mixture containing 5 µl of 2X Pushion blood PCR Buffer, 0.15 µl of Phusion blood DNA polymerase, 0.25 µM of each primer and 1 µl of the product of first amplification. All amplifications were performed by denaturing at 98 °C for 5 minutes, followed by 35 cycles of 98 °C for 1 second, 62 °C for 5 seconds, and 72 °C for 35 seconds, before a final extension at 72 °C for 5 minutes. The products of the nested PCR reactions were separated in 1.5 % agarose gels and visualized using the Syngene Sydr2/1436 Chemigenius 2 Bio Imaging System Darkroom W/ Syngene Gelvue.

### Detection of *Pfhrp2* and *Pfhrp3* genes

The presence of *Pfhrp2* and *Pfhrp3* genes was evaluated by PCR amplification. *Pfhrp2* and *Pfhrp3* genes were amplified using a nested PCR approach as previously reported^33,70^. Briefly, thirty randomly selected 18S PCR amplification positive samples were amplified using primers spanning fragments of exons 1 and 2 and intervening intron for *Pfhrp2* and *3*. Genes immediately upstream and downstream of *Pfhrp2* (PF3D7_0831900 and PF3D7_0831700 respectively) and *Pfhrp3* (PF3D7_1372100 and PF3D7_1372400 respectively) were amplified using the primers and PCR conditions as indicated in Gamboa *et al*., and Dorado *et al*.^33,35^.

### Drug resistance loci genotyping

Four known drug resistance loci were amplified from the extracted DNA by nested PCR, focussing on regions known to contain resistance mutations: *Pfkelch13* propeller domain (PF3D7_1343700), *Pfdhfr* (amino acid positions: 16, 50, 51, 59, 108, 140, and 164), *Pfdhps* (amino acid positions: 436, 437, 540, 581, and 613) and *Pfcrt* (amino acid positions: 72 to 76). Primer information is provided in Table S1 4, and amplification conditions were as follows: *Pfkelch13*: First PCR: 1 × $9{5}^{\circ }$C 15 min; 30 × ($9{5}^{\circ }$C 0.5 min, 58 ^o^C 2 min, $7{2}^{\circ }$C 2 min); 1 × $7{2}^{\circ }$C 10 min. Nested PCR: 1 × $9{5}^{\circ }$C 10 min; 40 × ($9{5}^{\circ }$C 0.5 min, $6{2}^{\circ }$C 1 min, $7{2}^{\circ }$C 1min); 1 × $7{2}^{\circ }$C 10 min. *Pfdhfr*: First PCR: 1 × $9{4}^{\circ }$C 3 min; 40 × ($9{4}^{\circ }$C 1 min, $5{2}^{\circ }$C 2 min, $7{2}^{\circ }$C 1 min); 1 × $7{2}^{\circ }$C 10 min. Nested PCR: 1 × $9{4}^{\circ }$C 3 min; 4 × ($9{4}^{\circ }$C 1 min, $4{6}^{\circ }$C 2 min, $7{2}^{\circ }$C 1 min); 34 × ($9{4}^{\circ }$C 1 min, $4{5}^{\circ }$C 1 min, $7{2}^{\circ }$C 1 min); 1 × $7{2}^{\circ }$C 10 min. *Pfdhps*: First PCR: 1 × $9{4}^{\circ }$C 3 min; 40 × ($9{4}^{\circ }$C 1 min, $5{1}^{\circ }$C 2 min, $7{2}^{\circ }$C 1min); 1 × $7{2}^{\circ }$C 10 min. Nested PCR: 1 × $9{4}^{\circ }$C 3 min; 40 × ($9{4}^{\circ }$C 1 min, $5{2}^{\circ }$C 2 min, $7{2}^{\circ }$C 1 min); 1 × $7{2}^{\circ }$C 10 min. *Pfcrt*: First PCR: 1 × $9{4}^{\circ }$C 3 min; 45 × ($9{4}^{\circ }$C 0.5 min, $5{6}^{\circ }$C 0.5 min, $6{0}^{\circ }$C 1 min); 1 × $6{0}^{\circ }$C 3 min. Nested PCR: 1 × $9{5}^{\circ }$C 5 min; 30 × ($9{2}^{\circ }$C 0.5 min, $4{8}^{\circ }$C 0.5 min, $6{5}^{\circ }$C 1 min); 1 × $6{5}^{\circ }$C 3 min. The amplified products were Sanger dideoxy sequenced (Macrogen) with primers used in nested reaction. 87 samples were sequenced for *Pfcrt*, 87 for *Pfdhfr*, 74 for *Pfdhps* and 230 for *PfKelch13*. Sequences were aligned using MEGA 7.0.26^71^ against reference 3D7 sequence for each gene and mutations were identified.

### Microsatellite and SNP genotyping

Microsatellite genotyping was carried out on 153 positive samples diagnosed at the malaria posts: 103 from Guapi (Guapi, Cauca), 7 from Carmelo (Guapi, Cauca), 14 from Chanzará (Santa Bárbara de Iscuandé, Nariño) and 29 from Cuerval (Timbiquí, Cauca). Six neutral microsatellite markers distributed across six chromosomes TA1 (chr. 6), TA109 (chr. 6), PfPk2 (chr. 12), Poly$\alpha$ (chr. 4), C3M69 (chr. 3) and C2M34 (chr 2), were amplified and genotyped as previously described^27,61^. Fluorescently labelled primers (HEX and FAM) were used to amplify microsatellite alleles and size differentiation was performed by capillary electrophoresis on an ABI 3130xl sequencer (Applied Biosystems, USA) using Rox350 as molecular weight marker. Allele sizes were scored using GeneMapper version 5 (Applied Biosystems, USA) and Genemarker beta version (Softgenetics), using the default settings for microsatellite analysis. Each electropherogram was manually explored and allele sizes were normalized with respect to controls. The peaks with relative fluorescent units (FRU) below 100 were excluded from analysis as well as samples with two or more peaks. SNP genotyping and estimates of the Complexity of Infection were obtained through SpotMalaria Genetic Report Card from MalariaGen (https://www.malariagen.net/projects/spotmalaria) from 207 samples collected at the malaria posts located in Guapi (172) and El Cuerval (35). Briefly, purified DNA was initially amplified in multiplex using locus-specific primers and subsequently, to identify a SNP, a second amplification was performed with single base extension PCR using SNP specific primers. To account for product overlaps in molecular weight four reactions were performed separately. PCR products were analysed by mass spectrometry on Agena 384 well array chips and SNP calls made using automated MalariaGEN pipelines. In total, 101 positions across the *P. falciparum* genome were analyzed for polymorphisms in this manner. Since the SNP barcode dataset contained both loci and samples with missing calls, we tested a number of combinations where we excluded loci with missing data at more than 10–40% of sites and/or samples missing above 5–10% of sites. Samples with multiple infections (heterozygous positions) were also excluded from the analysis. We used the most stringent dataset (barcode excluding above 10 percent of missing site per loci and 5 percent of missing sites per sample) as input for population structure inference.

### Population structure analysis

Population structure was determined through Bayesian inference using STRUCTURE version 2.3.4 software^72^. The STRUCTURE parameters used were as follows: an admixture model with correlated allele frequencies, a 100,000 burn-in period followed by 100,000 Markov Chain Monte Carlo (MCMC) repetitions for 10 iterations. The hypothesis of 2–10 likely clusters (K) corresponding to each diagnostic site or to a region of origin was tested. The most likely value of K for this population was inferred using the Evanno method as implemented in the STRUCTURE Harvester program^73^. Principal component analysis (PCA) was performed using R version 3.5.2 with the integrated function prcomp, that uses a singular value decomposition of the data matrix, on the 49 SNPs for 117 samples obtained from the SpotMalaria Genetic Report Card.

### Clonality of infections

Genetic distances were calculated as all proportional pairwise differences (p-distances) from an alignment of barcode 10-5 and clonality was assessed by generating a UPGMA tree using p-distances as implemented in MEGA 7.0.26^71^. Distances and diversity were calculated using MEGA 7.0.26. Clonality was defined as the subpopulation of parasites having mean distance within populations equal to 1% or less. Multiplicity of Infection (MOI) was estimated using the Real McCOIL^74^ as reported by Gene Report Card. Files generated by STRUCTURE were visualized using pophelper 2.2.8.1 (royfrancis.github.io/pophelper/).

### Topological data analysis

The spatial and temporal distribution of genotypes was analysed using our own implementation of the Mapper algorithm^75^ based on the existing R package: *TDAmapper*^76^ (Supplementary Material 2). This algorithm takes as input a finite metric space and a filter function and outputs a graph. Genetic distance (p-distances) derived from the SNP barcode (Barcode 10-5) and calculated in MEGA7 were taken as the finite metric space. The filter function assigns to each case the elapsed time of the case since the beginning of the study. Hierarchical clustering was used in the implementation of the Mapper algorithm to assign cases to nodes. For georeferenced data each node of the Mapper graph is a cluster of cases plotted on a map and connecting all cases that share a cluster. This results in a series of “cliques” (i.e. a set of nodes that are all connected to each other) that are interconnected by the edges of Mapper 1-skeleton graph. Note that the Mapper algorithm can assign two genotypes that STRUCTURE identified within a single subpopulation to separate clusters, thus resulting in multiple clusters of the same colour within one interval in the 1-skeleton. This happens because clustering algorithms detect high distances within the interval between the two genotypes, given all the cases of the interval. Connections are simultaneously plotted as an epidemic curve (cases over time) for each sub-population and can be observed as an animated visualisation (Supplemental Animation 1). Genotypes were also visualised on the map and each genotype connected to the genotype with highest in-degree centrality in the cluster where it belongs. The nodes with high centrality are those which connect different clusters, i.e. the overlapping intervals in the Mapper algorithm. This results in a graph that highlights connections among clusters over the whole study period, more precisely, the Point Intersection Network of the TDA 1-skeleton graph. We also aggregated this case network by geographic location by contracting edges between cases with the same location. Descriptive network statistics such as Pagerank and betweenness centrality (definitions included in Supplementary Material 2) were calculated for both case and geographic networks, to study the relative importance of each case and location. For the purpose of verification of our results, we implemented a different TDA method to confirm our findings. The results of performing persistent homology analysis are discussed in the TDA methodological appendix in the supplementary material.

## Supplementary Information

### Supplementary Information

Supplementary Information.

### Supplementary Information

Supplementary Information 2

## Data Availability

The dataset generated during and/or analysed during the current study are available from the corresponding author on reasonable request.
