# Supplementary material for: Spatio-temporal dynamics of Plasmodium falciparum transmission within a spatial unit on the Colombian Pacific Coast
Source: Sci Rep. 2020 Feb 28;10:3756. doi: 10.1038/s41598-020-60676-1 (PMC7048816; doi:10.1038/s41598-020-60676-1)
Supplement: Supplementary file 1 — Supplementary Information. [file 41598_2020_60676_MOESM1_ESM.pdf]

Supplement 1: Spatio-temporal dynamics of *Plasmodium falciparum* transmission within a spatial unit on the Colombian Pacific Coast

Angélica Knudson[1], Felipe González-Casabianca[9,10], Alejandro Feged-Rivadeneira[10,*], Maria Fernanda Pedreros[2], Samanda Aponte[2], Adriana Olaya[3], Carlos F. Castillo[3], Elvira Mancilla[3], Anderson Piamba-Dorado[3], Ricardo Sanchez-Pedraza[4], Myriam Janeth Salazar-Terreros[5], Naomi Lucchi[6], Venkatachalam Udhayakumar[6], Chris Jacob[7], Alena Pance[7], Manuela Carrasquilla[12], Giovanni Apráez[2,3], Jairo Andrés Angel[9,11], Julian C. Rayner[8,*], and Vladimir Corredor[2,*]

[1]Departamento de Microbiología, Facultad de Medicina, Universidad Nacional de Colombia, Bogotá, Colombia
[2]Departamento de Salud Pública, Facultad de Medicina, Universidad Nacional de Colombia, Bogotá, Colombia
[3]Secretaría Departamental de Salud del Cauca, Popayán, Colombia
[4]Departamento de Psiquiatria, Facultad de Medicina, Universidad Nacional de Colombia
[5]Post-doctoral fellow, Centro de Hematologia e Hemoterapia (HEMOCENTRO), Universidade Estadual de Campinas (UNICAMP) Brazil.
[6]Malaria Branch, Division of Parasitic Diseases and Malaria, Centers for Global Health, Centers for Disease Control and Prevention, Atlanta, 30030, GA United States of America
[7]Malaria Programme, Wellcome Sanger Institute, Wellcome Genome Campus, Cambridge CB10 1SA, United Kingdom
[8]Cambridge Institute for Medical Research, University of Cambridge, Cambridge CB2 0XY, United Kingdom
[9]Departamento de Matemáticas, Facultad de Ciencias, Universidad de Los Andes, Bogotá, Colombia
[10]Gestión y desarrollo urbanos, Facultad de Ciencia Política, Universidad del Rosario, Bogotá, Colombia
[11]Department of Mathematics and Statistics, Universidad del Norte, Barranquilla, Colombia
[12]Department of Immunology and Infectious Diseases, Harvard T.H. Chan School of Public Health, Boston, Massachusetts 02115
[*]Correspondence and request for materials should be addressed to V.C. (vcorredore@unal.edu.co) or J.C.R (jcr1003@ac.uk) or A.F-R. (alejandro.feged@urosario.edu.co).

ABSTRACT

This supplement includes information describing the context of our findings. Detailed information by origin, time, and other variables is presented in Tables S1 1-S1 4. Figure S1 1 shows cases for the Pacific Coast of Colombia, and the Guapi municipality (data from SIVIGILA, the official public health surveilance system). Figure S1 2 shows allele frequencies for loci associated with drug resistance. Map S1 3 shows the spatial distribution of parasite subpopulations in the area of study. Map S1 4 represent the suggested malaria transmission unit.

Figures

	MALARIA CASES (P.falciparum) DIAGNOSED IN GUAPI				
	**2014**	**2015**	**2016**	**2017**	**TOTAL**
**GUAPI URBAN (Neighborhood)**					
20 de Julio	1 (0.3)	0	0	0	1
Batallón	1 (0.3)	0	0	0	1
Bellavista	2 (0.5)	0	9 (2.4%)	7 (1.9)	18
Canaan	0	0	0	0	0
El Carmen	0	1 (0.3)	1 (0.3)	9 (2.4%)	11
Fortaleza	5 (1.3)	1 (0.3)	1 (0.3)	0	7
Jardin	0	0	2 (0.5)	1 (0.3)	3
Las Flores	2 (0.5)	0	0	0	2
Olimpico	0	3 (0.8)	1 (0.3)	4 (1.1)	8
San Martin	0	0	1 (0.3)	0	1
Santa Mónica	8 (2.2)	7 (1.9)	2 (0.5)	0	17
Venecia	0	0	1 (0.3)	0	1
**Total urban cases**	**19 (70.4)**	**12 (37.5)**	**18 (9.7)**	**21 (16.5)**	**70 (18.8)**
**GUAPI RURAL**					
Bagrero, Narino	1 (3.7)	3 (9.4)	6 (3.2)	0	10
Bella Vista, Chanzará, Nariño	0	0	1 (0.5)	0	1
Bocas de Satinga, Nariño	0	1 (3.1)	0	0	1
Buena Vista, Timbiquí, Cauca	1 (3.7)	0	0	0	1
Calle Honda, Guapi	0	4 (12.5)	0	0	4
Carmelo, Guapi, Cauca	0	0	18 (9.7)	51 (13.7)	69
Chamón, Guapi, Cauca	0	0	5 (2.7)	1 (0.3)	6
Chanzará, Nariño	1 (3.7)	3 (9.4)	9 (4.8)	3 (0.8)	16
Chico Perez, Nariño	1 (3.7)	2 (6.3)	9 (4.8)	2 (0.5)	14
Comer, Guapi, Cauca	0	0	0	1 (0.3)	1
Cuerval, Timbiquí	0	0	1 (0.5)	0	1
El Charco, Nariño	0	1 (3.1)	2	0	3
Iscuandé, Nariño	1 (3.7)	0	0	0	1
Juanico, Guapi, Cauca	0	0	1 (0.5)	1 (0.3)	2
Las Parcelas, Guapi, Cauca	0	0	0	1 (0.3)	1
Las Peñas, Chanzará, Nariño	0	0	1 (0.5)	1 (0.3)	2
Las Varas, Guapi, Cauca	0	0	0	1 (0.3)	1
Limones, Guapi, Cauca	1 (3.7)	0	77	12 (3.23)	90
Loro, Juajui, Guapi, Cauca	0	0	3	1 (0.3)	4
Madrid, Iscuandé, Nariño	0	0	1 (0.5)	0	1
Patía, Nariño	1 (3.7)	0	0	2 (0.5)	3
Playa Chacón, Timbiquí, Cauca	0	1 (3.1)	2	0	3
Quiroga, Guapi, Cauca	0	0	25	7 (1.9)	32
San José de Guare, Guapi, Cauca	0	1 (3.1)	0	19 (5.1)	20
San Miguel del Mar, Timbiquí, Cauca	0	0	1 (0.5)	0	1
San Pedro, Nariño	0	0	1 (0.5)	0	1
Santa Rosa, Guapi, Cauca	0	0	1 (0.5)	1 (0.3)	2
Satinga, Nariño	0	0	0	2 (0.5)	2
Temuey, Guapi, Cauca	1 (3.7)	0	1 (0.5)	0	2
Timbiquí, Cauca	0	0	1 (0.5)	0	1
Tierra Firme, Iscuandé, Nariño	0	2 (6.3)	0	0	2
Vuelta Larga, Guapi, Cauca	0	0	1 (0.5)	0	1
Mining area,, Venezuela	0	2 (6.3)	1 (0.5)	0	3
**Total rural cases**	**8 (29.6)**	**20 (62.5)**	**168 (90.3)**	**106 (83.5)**	**302 (81.2)**
**Total cases**	**27 (100)**	**32 (100)**	**186 (100)**	**127 (100)**	**372 (100)**

Supplemental Table S1 1. Origin and number of malaria cases diagnosed at the public malaria post during the study period (2014-2017). In parenthesis percentages per total number of cases per year.

Sampling (N)	April 2015 Santa Mónica	October 2015. Santa Mónica	April 2016. Santa Mónica	October 2016. Santa Mónica	December 2015. El Cuerval	May 2017. El Cuerval
**Female (%)**	110/226 (48.7)	127/250 (50.8)	152/298 (51)	89/188 (47.3)	152/298 (51)	89/188 (47.3)
**Ethnicity (%)**						
**Afro-Colombian**	222 (98.2)	248 (99.2)	290 (97.3)	188 (100)	290 (97.3)	188 (100)
**Mixed**	2 (0.9)	2 (0.8)	8 (2.7)	0	8 (2.7)	0
**White**	2 (0.9)	0	0	0	0	0
**Age (years)**	3 a 94	3 a 94	3 a 93	2 a 74	3 a 93	2 a 74
**Time of Residence (years)**	0.1-40	0.1-45	0.2-9	0.1-9	0.2-9	0.1-9
**Health System (%)**						
**Contributive-based healthcare**	24 (11)	17 (6.9)	27 (9.2)	8 (4.37)	27 (9.2)	8 (4.37)
**Subsidised Healthcare**	176 (80.7)	202 (82.5)	246 (84)	148 (80.9)	246 (84)	148 (80.9)
**Bound to healtcare system + other + unknown**	0	2 (0.8)	4 (1.4)	6 (3.3)	4 (1.4)	6 (3.3)
**No Health card**	18 (8.3)	23 (9.4)	15 (5.1)	21 (11.5)	15 (5.1)	21 (11.5)
**Other**	0	1 (0.4)	0	0	0	0
**Not known**	0	0	1 (0.3)	0	1 (0.3)	0
**Occupation (%)** **Student**	118 (53.9)	120 (48.0)	121 (40.7)	76 (40.9)	121 (40.7)	76 (40.9)
**Housewife**	23 (10.5)	42 (16.8)	51 (17.2)	30 (16.1)	51 (17.2)	30 (16.1)
**Fisherman**	5 (2.3)	6 (2.40)	10 (3.4)	42 (22.6)	10 (3.4)	42 (22.6)
**Other**	73 (33.3)	82 (29.2)	115 (38.7)	38 (20.4)	115 (38.7)	38 (20.4)

Supplemental Table S1 2. Socio-demographic data relating to asymptomatic individuals identified in Santa Mónica, Guapi and El Cuerval (Timbiquí municipality).

SAMPLE ID	LOCALITY	*PF3D7*_0831900	*HRP2* (chr8) (*PF3D7*_0831800)	*PF3D7*_0831700	*PF3D7*_1372100	*HRP3* (chr13)*PF3D7*_1372200	*PF3D7*_1372400
3D7		+	+	+	+	+	+
Dd2		-	-	-	+	+	+
HB3		+	+	+	-	-	-
GU013	Unknown, Guapi, Cauca	+	+	+	ND	-	+
GU025	Sta.Mónica, Guapi, Guapi, Cauca	+	+	+	-	-	+
GU031	Unknown, Guapi, Cauca	+	+	+	-	-	+
GU035	Sta.Mónica, Guapi, Guapi, Cauca	+	+	+	-	-	+
GU036	Fortaleza, Guapi, Guapi, Cauca	+	+	+	-	-	+
GU037	Unknown, Guapi, Cauca	+	+	+	+	+	+
GU040	Temuey, Guapi, Cauca	+	+	+	-	-	-
GU043	Patía, Nariño	+	+	+	-	-	+
GU053	Unknown, Guapi, Cauca	+	+	+	-	-	+
GU058	Sta.Mónica, Guapi, Guapi, Cauca	+	+	+	-	-	+
GU059	Unknown, Guapi, Cauca	+	+	+	-	-	-
GU062	Sta.Mónica, Guapi, Guapi, Cauca	+	+	+	-	-	-
GU063	Unknown, Guapi, Cauca	+	+	+	-	-	+
GU064	Unknown, Guapi, Cauca	+	+	+	-	-	+
GU066	Chanzará, Nariño	+	+	+	-	-	+
GU067	Unknown, Guapi, Cauca	+	+	+	-	-	+
GU069	Unknown, Guapi, Cauca	+	+	+	-	-	+
GU072	Unknown, Guapi, Cauca	+	+	+	-	-	+
GU073	Unknown, Guapi, Cauca	+	+	+	-	-	+
GU074	Unknown, Guapi, Cauca	+	+	+	-	-	+
GU077	Bagrero, Nariño	+	+	+	-	-	-
GU081	Unknown, Guapi, Cauca	+	+	+	-	-	-
GU082	Bocas de Satinga, Nariño	+	-	+	-	-	-
GU083	Unknown, Guapi, Cauca	+	+	+	+	-	-
GU089	Unknown, Guapi, Cauca	+	+	+	-	+	+
CU009	Cuerval, Timbiquí, Cauca	+	-	+	-	-	-
CU010	Cuerval, Timbiquí, Cauca	+	+	+	-	-	+
CU011	Cuerval, Timbiquí, Cauca	+	+	+	-	-	+
GU037	Unknown, Guapi, Cauca	+		+	+		+
GU050	Zona Minera, Venezuela	+	+	+	-	-	-

Supplemental Table S1 3. Deletions of the *Pfhrp2*, *Pfhrp3* and flanking genes in *P.falciparum* samples diagnosed at the Guapi Malaria Post.

PfKelch13 (Propeller domain)		
**Primer name**	**PCR**	**Sequence (5’ - 3’)**
**K13_PCR_ forward**	First PCR	CGGAGTGACCAAATCTGGGA
**K13_PCR_ reverse**		GGGAATCTGGTGGTAACAGC
**K13_N1_ forward**	Nested 1	GCCAAGCTGCCATTCATTTG
**K13_N1_ reverse**		GCCTTGTTGAAAGAAGCAGA
**K13_N2_ forward**	Nested 2	CGCCAGCATTGTTGACTAAT
**K13_N2_ reverse**		GCGGAAGTAGTAGCGAGAAT
Pfdhfr		
**Primer name**	**PCR**	**Sequence (5’ - 3’)**
**M1_Outer_forward**	First PCR	TTT ATG ATG GAA CAA GTC TGC
M7_Outer_ reverse		CTA GTA TAT ACA TCG CTA ACA
**M3b_Inner_ forward**	Nested	TGA TGG AAC AAG TCT GCG ACG
**M9_Inner_ reverse**		CTG GAA AAA ATA CAT CAC ATT
Pfdhps		
**Primer name**	**PCR**	**Sequence (5’ - 3’)**
**N1_Outer_ forward**	First PCR	GATTCTTTTTCAGATGGAGG
**N2_Outer_ reverse**		TTCCTCATGTAATTCATCTGA
**R2_Inner_ forward**	Nested	AACCTAAACGTGCTGTTCAA
**R_Inner_ reverse**		AATTGTGTGATTTGTCCACAA
Pfcrt		
**Primer name**	**PCR**	**Sequence (5’ - 3’)**
**CRTP1 forward**	First PCR	CCGTTAATAATAAATACACGCAG
**CRTP2 reverse**		CGGATGTTACAAAACTATAGTTACC
**CRTD1 forward**	Nested	TGTGCTCATGTGTTTAAACTT
**CRTD2 reverse**		CAAAACTATAGTTACCAATTTTG

Supplemental Table S1 4. Primer sequence.

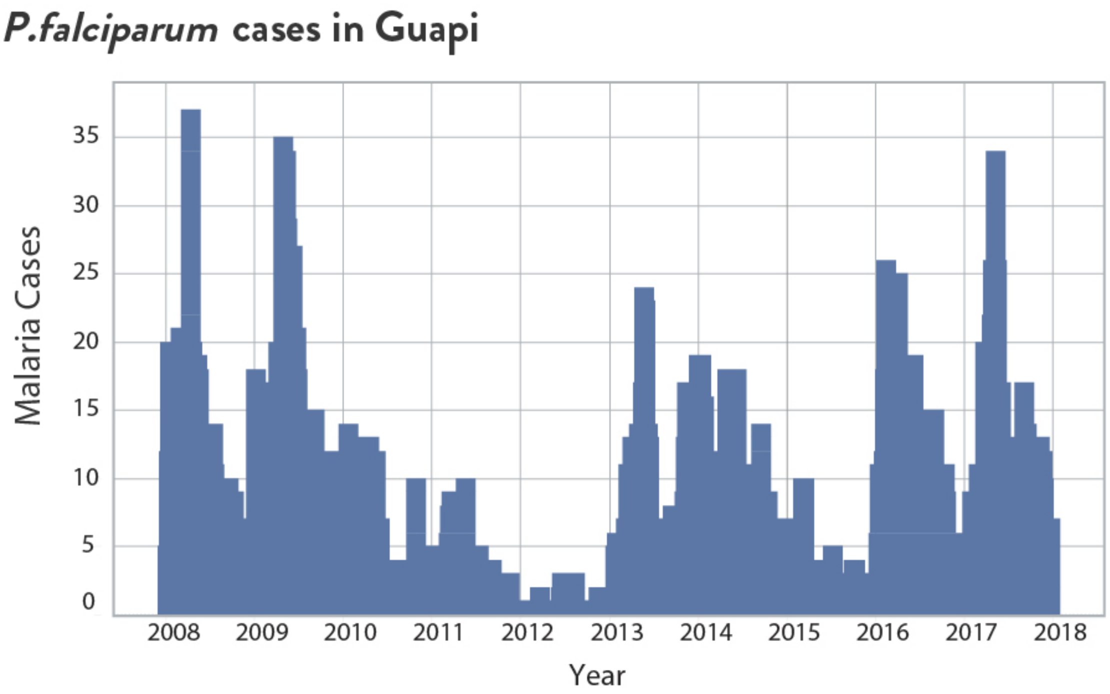

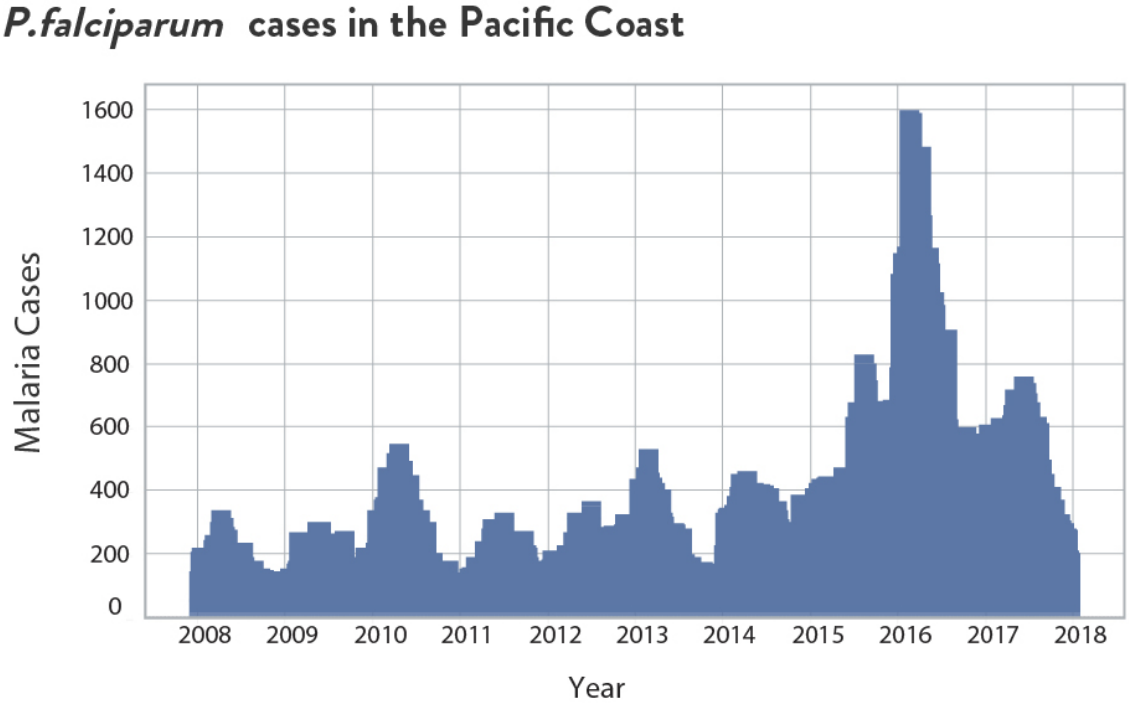

Supplemental Figure S1 1. Epidemic curves for Guapi and the Pacific Coast. Note that while infection by *P.falciparum* in the Pacific Coast have increased in intensity over the past 10 years, Guapi has experienced outbreaks of similar magnitudes over the same period.

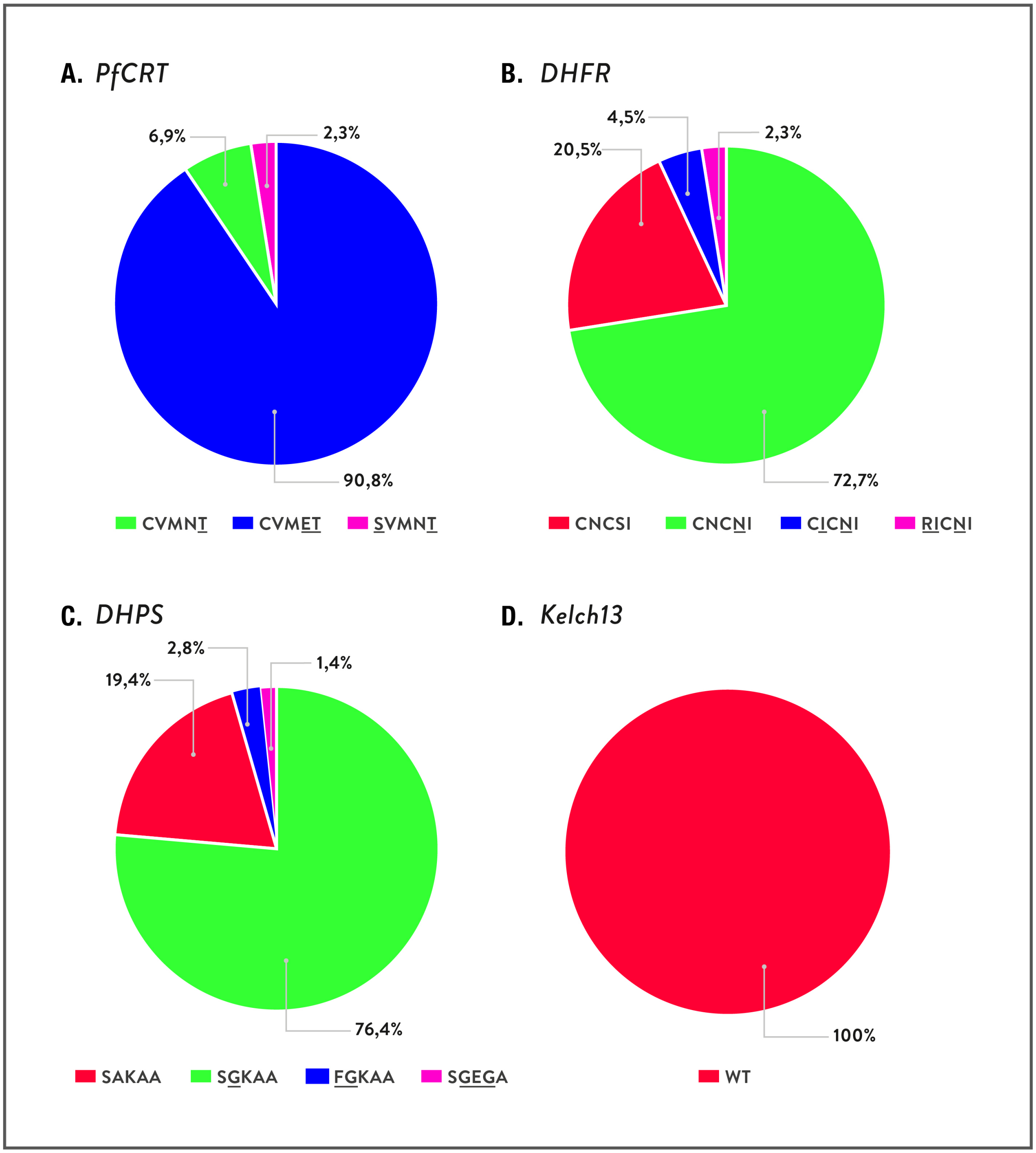

Supplemental Figure S1 2. Allele frequencies for *Pfcrt* (positions 72-76), *dhfr* (positions 50, 51, 59, 108, 164), *dhps* (positions 436, 437, 540, 581, 613) and *kelch13* (propeller domain) in samples diagnosed in Guapi, Cauca between 2014 and 2017. Mutant positions underlined

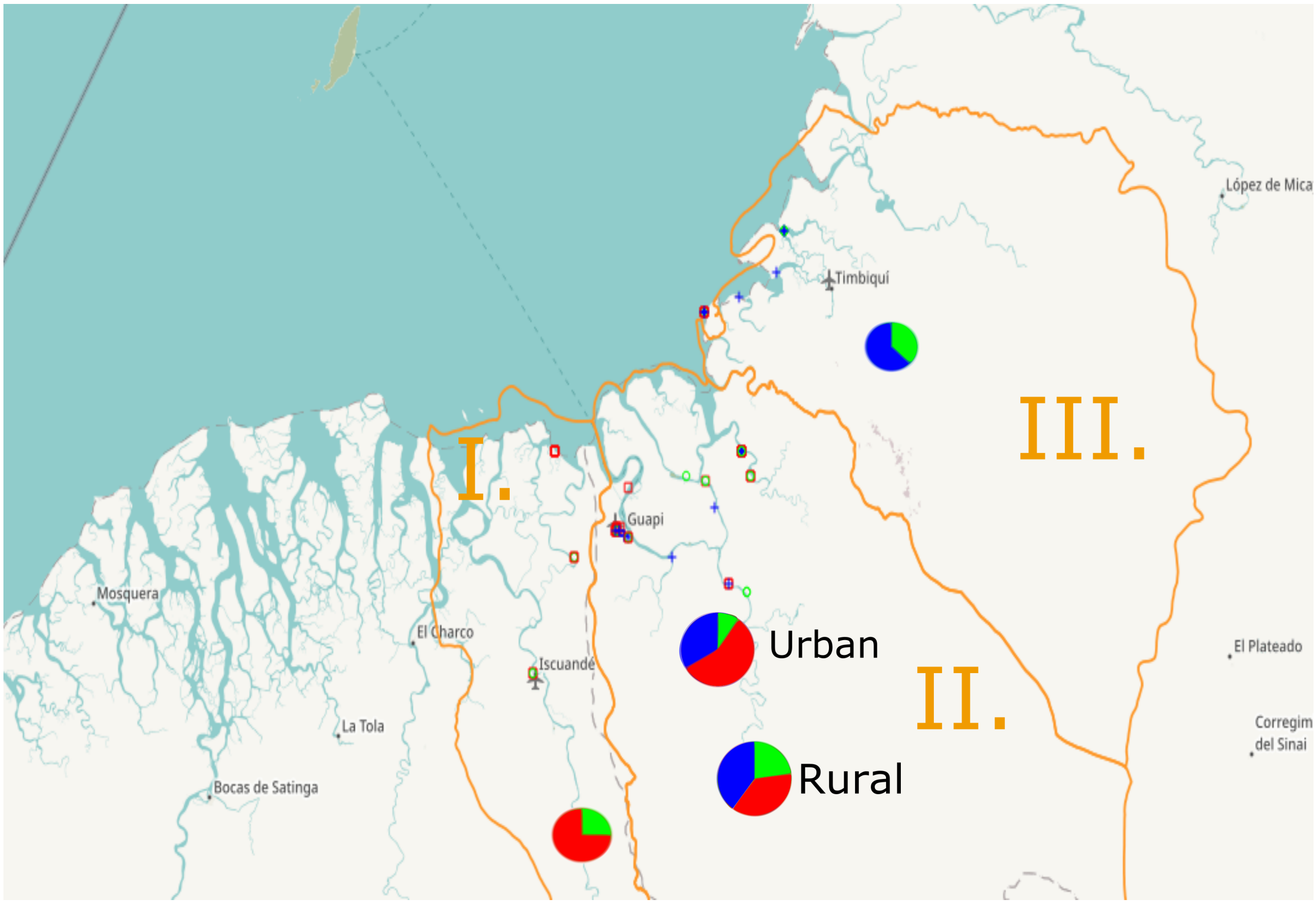

Supplemental Figure S1 3. Distribution of *P.falciparum* populations identified by STRUCTURE (Green: population A, Red: population B; Blue: population C). I, II and III indicate the Santa Bárbara de Iscuandé, Guapi and Timbiquí municipalities respectively. Pie charts indicate the frequency of each population. II and III are connected by riverways. Note that cases may overlap. QGIS version 2.18 (https://www.qgis.org)[1]. Source: OpenStreetMap[2], © OpenStreetMap contributors, under an Attribution-Share-Alike 2.0 Generic licence (https://creativecommons.org/licenses/by-sa/2.0/).

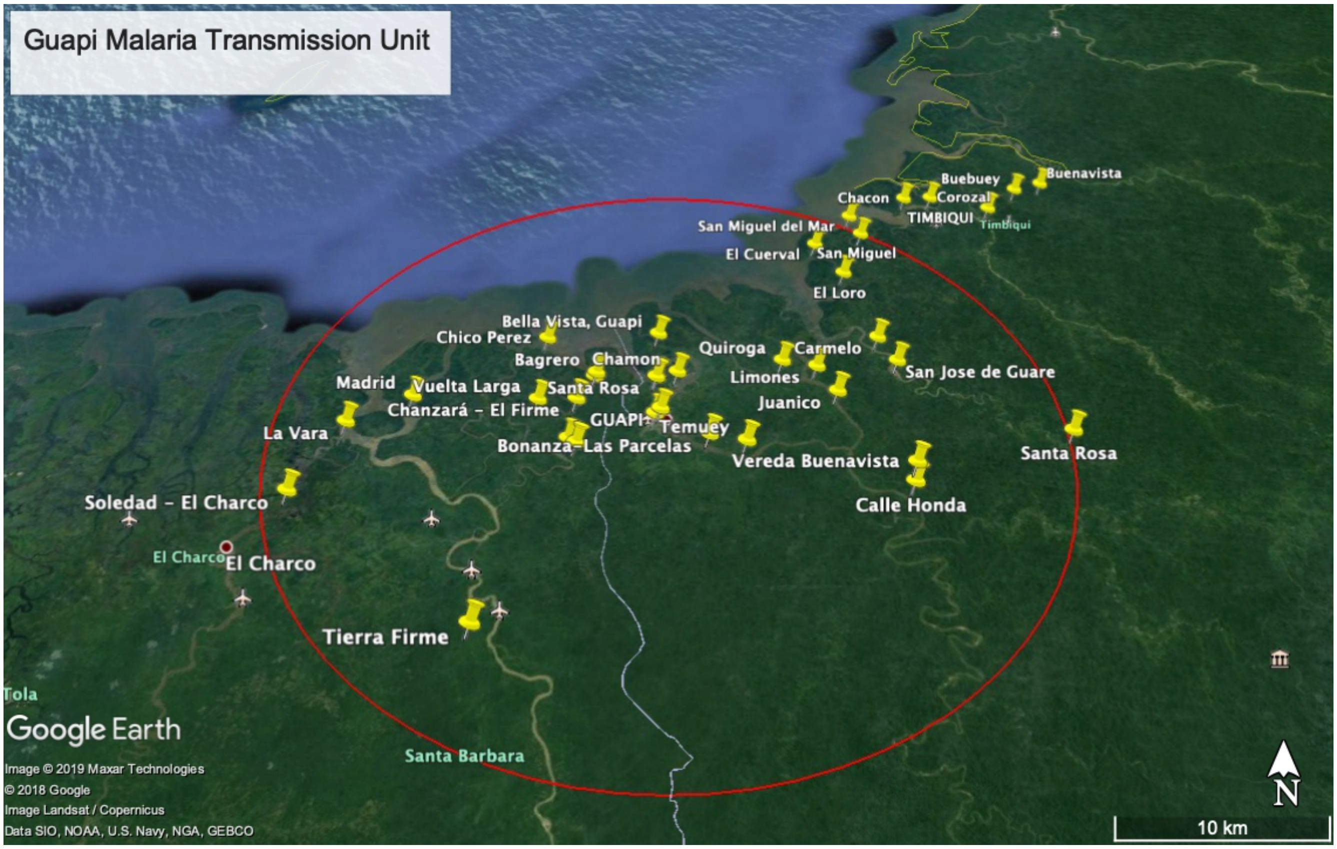

Supplemental Figure S1 4. Guapi Malaria Transmission Unit. Sites of origin of malaria cases diagnosed at the Public Malaria Post in Guapi between 2014-2017. Red circle indicates Malaria Transmission Unit (i.e. area of origin of 95% or more of the malaria cases

References

1. QGIS Development team (2018). Quantum GIS Geographic Information System. Open Source Geospatial Foundation Project. http://qgis.osgeo.org .
2. OpenStreetMap contributors. Planet dump retrieved from https://planet.osm.org . https://www.openstreetmap.org, 2017.
