# Supplementary material for: Spatio-temporal dynamics of Plasmodium falciparum transmission within a spatial unit on the Colombian Pacific Coast
Source: Sci Rep. 2020 Feb 28;10:3756. doi: 10.1038/s41598-020-60676-1 (PMC7048816; doi:10.1038/s41598-020-60676-1)
Supplement: Supplementary file 2 — Supplementary Information 2 [file 41598_2020_60676_MOESM2_ESM.pdf]

Supplement 2: Spatio-temporal dynamics of *Plasmodium falciparum* transmission within a spatial unit on the Colombian Pacific Coast

Angélica Knudson[1], Felipe González-Casabianca[9,10], Alejandro Feged-Rivadeneira[10,*], Maria Fernanda Pedreros[2], Samanda Aponte[2], Adriana Olaya[3], Carlos F. Castillo[3], Elvira Mancilla[3], Anderson Piamba-Dorado[3], Ricardo Sanchez-Pedraza[4], Myriam Janeth Salazar-Terreros[5], Naomi Lucchi[6], Venkatachalam Udhayakumar[6], Chris Jacob[7], Alena Pance[7], Manuela Carrasquilla[12], Giovanni Apráez[2,3], Jairo Andrés Angel[9,11], Julian C. Rayner[8,*], and Vladimir Corredor[2,*]

[1]Departamento de Microbiología, Facultad de Medicina, Universidad Nacional de Colombia, Bogotá, Colombia
[2]Departamento de Salud Pública, Facultad de Medicina, Universidad Nacional de Colombia, Bogotá, Colombia
[3]Secretaría Departamental de Salud del Cauca, Popayán, Colombia
[4]Departamento de Psiquiatria, Facultad de Medicina, Universidad Nacional de Colombia
[5]Post-doctoral fellow, Centro de Hematologia e Hemoterapia (HEMOCENTRO), Universidade Estadual de Campinas (UNICAMP) Brazil.
[6]Malaria Branch, Division of Parasitic Diseases and Malaria, Centers for Global Health, Centers for Disease Control and Prevention, Atlanta, 30030, GA United States of America
[7]Malaria Programme, Wellcome Sanger Institute, Wellcome Genome Campus, Cambridge CB10 1SA, United Kingdom
[8]Cambridge Institute for Medical Research, University of Cambridge, Cambridge CB2 0XY, United Kingdom
[9]Departamento de Matemáticas, Facultad de Ciencias, Universidad de Los Andes, Bogotá, Colombia
[10]Gestión y desarrollo urbanos, Facultad de Ciencia Política, Universidad del Rosario, Bogotá, Colombia
[11]Department of Mathematics and Statistics, Universidad del Norte, Barranquilla, Colombia
[12]Department of Immunology and Infectious Diseases, Harvard T.H. Chan School of Public Health, Boston, Massachusetts 02115
[*]Correspondence and request for materials should be addressed to V.C. (vcorredore@unal.edu.co) or J.C.R (jcr1003@ac.uk) or A.F-R. (alejandro.feged@urosario.edu.co).

ABSTRACT

A methodological appendix is provided, containing detailed definitions of the terms employed throughout the main article in terms of Topological Data Analysis (TDA) and Social Network Analysis (SNA), as well as a review of the applications of TDA in population genetics, epidemiology and similar fields. An analysis of the same data-set with a different TDA method is also presented.

1 Topological data analysis

In this paper we used a tool derived from applied algebraic topology, the Mapper algorithm[1], to visualize the evolution of the populations over time. We also used a strategy[5] to study evolution beyond phylogenetic trees, that allows capturing clonal and reticulate evolution by means of representing clonal evolution through trees, having a trivial topology (although highly non-trivial combinatorics) and reticulate evolution displaying nontrivial topologies.

Apart from detecting reticulate evolution, it is possible to determine the rate of recombination. Chan, Carlsson and Rabadan[5] applied their methods to viral genomes due to the existence of rich data sets. Their framework is based on persistent homology, a tool from the field of applied algebraic topology.

In this appendix we followed their framework and applied persistent homology to parasite genetic data from Guapi providing more evidence on the existence of reticulate evolution.

Algebraic topology, a branch of mathematics, has been considered up to recently a purely theoretical subject, it studies

properties of objects that are invariant under continuous deformations. Examples, with these kind of properties, that do not change under deformations are the number of components, or the number of holes in a space. In fact, algebraic topology has seen spectacular results by developing sophisticated invariants and tools to study and measure high dimensional holes. A central problem of algebraic topology, and other areas of mathematics such as differential geometry, is to understand the relation between local and global features. Many of the tools of algebraic topology are designed to detect global properties while ignoring local properties.

In the past decades a new sub-field has emerged which aims at using the underlying shape of data. *Topological Data Analysis* (TDA), consists of a set of tools and algorithms based on ideas of algebraic topology that provide insights based on the shape of data. These novel methods provide robust, coordinate-free and stable tools to complement traditional data analysis and machine learning.

One of such tools, *Mapper*[1], constructs a summary of the data in the form of a graph, generating a representation of complex high dimensional data in a concise way. This graph allows for the analysis and visualisation of the information.

The vertices of the graph correspond to local clusters and the interactions between these clusters are encoded on the edges of the graph. It gives at the same time information about what happens at small scales (the local clusters and its features) and at large scales (the interaction of all local clusters giving rise to loops and flares).

Mapper is able to detect features at large and small scales better than other methods, such as principal component analysis (PCA) and cluster algorithms. In fact, it can be considered as a hybrid of dimensional reduction and clustering algorithms. Based on any dimensional reduction algorithm or on a set of relevant features on the data, a filter function is constructed. The projection of the data with this filter function is a first approximation at capturing a global behaviour. By covering the projection with overlapping bins and finding clusters in the original data in each bin, the local structure is revealed. Any clustering algorithm can be used and to each cluster a node is assigned. Clusters that share points are connected and the way these clusters interact forming loops and flares reveal the large scale behaviour.

Mapper has been used to find a subgroup of individuals with breast cancer that survived and was not discovered before[2,31], to study the voting behaviour in the congress of the USA[2], to find new types of basketball players in the NBA[2], to study pathogen persistence in soil[13], to discover novel patterns in spinal and brain injury[14], to find subgroups of individuals with different complications from type 2 diabetes[17], to study groups of patients of asthma[15],[16], to study infection cycles of mice and humans infected with the malaria parasite[18] and patterns of antibiotic resistance in pathogenic bacteria[9].

We will explain the mathematical basis of the *Mapper* algorithm in the following sections. Further details can be found elsewhere[2,3].

Persistence homology is another tool that offers remarkable applications: Persistent homology extracts the topological features of a filtered topological space and represents it with diagrams and barcodes. The barcodes provide information on the times when the topological features of the filtered space change.

Given some data with a notion of distance between the points, several spaces are constructed which are useful to study the data. These spaces are built by gluing points, lines, triangles, and higher-dimensional analogues and are presented mathematically as simplicial complexes.

Persistence homology has been applied to a number of fields, like the study of cancer[32],[33],[34], proteins[21],[22],[23],[24],[25], cell development[11],[12], robotics[26],[27],[28], signals in images[29],[30], periodicity in time series[20], phylogenetics[5],[6],[7],[8],[9] natural images[36], epidemiology[37],[38], materials science[39],[40],[41],[42] , networks in finance[43],[44], neuroscience[45],[46],[47],[48],[49],[50],[51],[52], classification of weighted networks[53], collaboration networks[54],[55], analysis of mobile data[56], biological aggregation models[57], time-series output of dynamical systems[58], natural-language analysis[59] and lung topology in chronic obstructive pulmonary disease[35].

1.1 Mathematical definitions

The aim of applied algebraic topology is to use the tools and techniques of algebraic topology to analyse high dimensional data. In many settings, from a collection of points that are thought to be sampled from an underlying space X, it is desirable to extract meaningful information from the points that capture geometric and topological information of X, by building a combinatorial representation of the data in terms of different structures: graphs and more general simplicial complexes.

Graphs

A *graph* G is a pair of sets (V,E), called the *vertices* (or *nodes*) and *edges* respectively. For every edge there is a pair of vertices. The graph is called simple if it has no loops (edges starting and ending on the same vertex) and no multiple edges between the same pair of vertices. If it has multiple edges or loops it is called a multigraph.

An *undirected graph* is a graph in which edges have no orientation, a directed graph is a graph in which edges have orientation. A *directed acyclic graph* is a directed graph with no directed cycles, i.e. there is no way to start at a vertex and follow directed edges and eventually go back to the same vertex.

Simplicial complexes

Definition 1 *A **simplicial complex** is a set K with a collection $S \subset \mathbb{P}(K)$, called **simplices**, such that:*

1. *If $k \in K$ then $\{k\} \in S$,*
2. *If $\tau \subset \sigma \in S$ then $\tau \in S$.*

*We call the singletons in S the **vertices of** K or 0-**simplices**. Also, we say that $\sigma \in S$ is a k-**simplex** if $|\sigma| = k+1$. Moreover, we will write each k-simplex $\sigma = [x_0, x_1, \dots, x_k]$ for $\{x_0, x_1, \dots, x_k\} \in S$. The **dimension of** K is $\sup\{|\sigma| \mid \sigma \in S\}$. The elements of S are called its simplicies. If $\sigma \in S$, the dimension of σ is $|\sigma| - 1$, the size of the set σ minus one. A simplex τ is a face of σ if τ is a subset of σ of a strictly smaller size.*

Definition 2 *A **sub-complex** of a simplicial complex K is a subset $L \subset K$ which is also a simplicial complex.*

Definition 3 *A **filtration** of a simplicial complex K is a nested subsequence of complexes*

$$\emptyset = K^0 \subset K^1 \subset \cdots \subset K^m = K.$$

We will define $K^i = K$ for any $i \geq m$.

In Figure S2 1 we present a filtration on a simplicial complex.

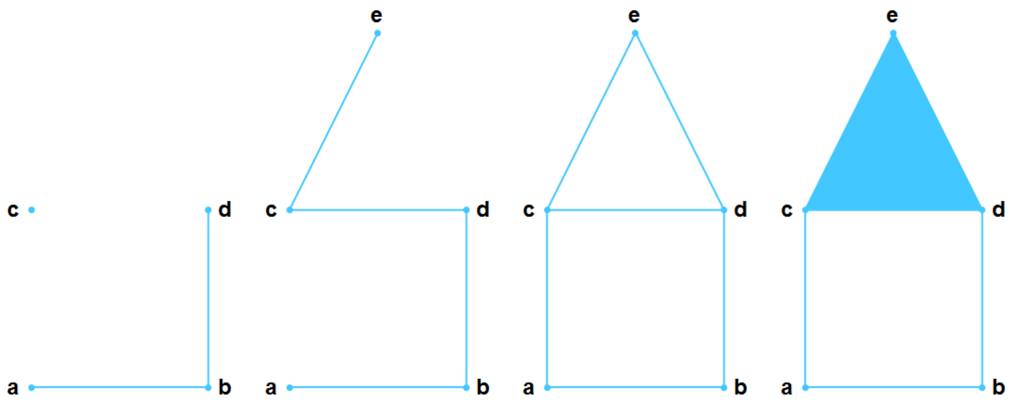

(a) K^1 **(b)** K^2 **(c)** K^3 **(d)** $K^4 = K$

Supplemental Figure S2 1. A filtration of the simplicial complex K.

Definition 4 (Clique complex) *Given a graph G there is a simplicial complex given by the **clique complex**: from a graph $G = (V, E)$, the clique complex associated to G is the simplicial complex X whose k-simplices X_k are sets of vertices of G of size $(k+1)$ such that every two vertices are adjacent, i.e. form a complete subgraph of G.*

Definition 5 (1-skeleton) *Given a simplicial complex X, the 1-**skeleton** is the undirected graph with vertices, the 0-simplices of X and the edges are the 1-simplices of X. It gives a 1-dimensional approximation of X ignoring the higher dimensional information.*

For applications to data science there are several simplicial complexes that are used.

Definition 6 (Nerve Complex) *Given a space X and a covering $\{U_\alpha\}_{\alpha \in \Delta}$ of X we define the **nerve complex**, as the the simplicial complex whose k-simplices are given by*

$$(N\{U_\alpha\}_{\alpha \in \Delta})_k := \left\{ \left[\{U_{\alpha_0}, \dots, U_{\alpha_k}\right] \mid U_{\alpha_0} \cap \ldots \cap U_{\alpha_k} \neq \emptyset \right\}.$$

i.e. given by the elements of the covering with non-empty intersection.

The nerve lemma states that when the covering $\{U_\alpha\}_{\alpha \in \Delta}$ is *good*, i.e every nonempty intersection is contractible, the nerve complex and the space X have the same homotopy type and thus the space X and the nerve complex have the same topological invariants.

One way to obtain coverings of a space is by using functions.

Definition 7 (Mapper complex) *Given a function $f : X \to \mathbb{R}$, the the mapper complex is the nerve complex of the cover obtained by the connected components of the cover given by overlapping intervals $\{I_n\}_{n\in\Delta}$ of the real numbers.*

Definition 8 (Čech complex) *Given a metric space (X,d) and $\varepsilon > 0$, the **Čech complex** is the simplicial complex given by the nerve complex of the covering of closed balls of radious ε.*

$$(C_\varepsilon(X))_k := \{[x_0,\dots,x_k] \mid \exists y \in X \; d(y,x_i) \leq \varepsilon \text{ for every } 1 \leq i \leq k\}.$$

Computationally it is very expensive to check for all simultaneous intersections of the balls and is easier to check only pairwise.

Definition 9 (Rips complex) *Given a metric space X and $\varepsilon > 0$. The **Rips complex** or **Vietoris-Rips complex** $\mathrm{R}_\varepsilon(X)$ is the simplicial complex whose k-simplices are given by*

$$(\mathrm{R}_\varepsilon(X))_k := \left\{[x_0,\dots,x_k] \mid x_i,x_j \in X, \; d(x_i,x_j) \leq \varepsilon \; \textit{for every} \; 1 \leq i,j \leq k\right\}.$$

When ε is sufficiently small the Vietoris-Rips complex has the same homotopy type of the Čech complex, but not in general. For every $\varepsilon > 0$, there is an inclusion of simplicial complexes:

$$C_\varepsilon \subseteq R_{2\varepsilon} \subseteq C_{2\varepsilon}$$

so any topological feature that persists for an interval of ε on the Čech complex has to persists also on the Rips complex.

Both the Čech and Rips complexes have the same underlying graph; indeed the Rips complex is the clique complex on that graph.

1.2 Persistent homology

The Čech and Rips complexes are filtered complexes, so it is possible to compute the topological features that persist when varying the distance scale ε.

Definition 10 *The **barcode** of a filtered simplicial complex K is a collection of horizontal line segments. These line segments are shown in a plane with the filtration parameter on the horizontal axes and an arbitrary ordering of the elements in the diagram as the vertical axes.*

In the following figure we show the barcodes for a filtered complex.

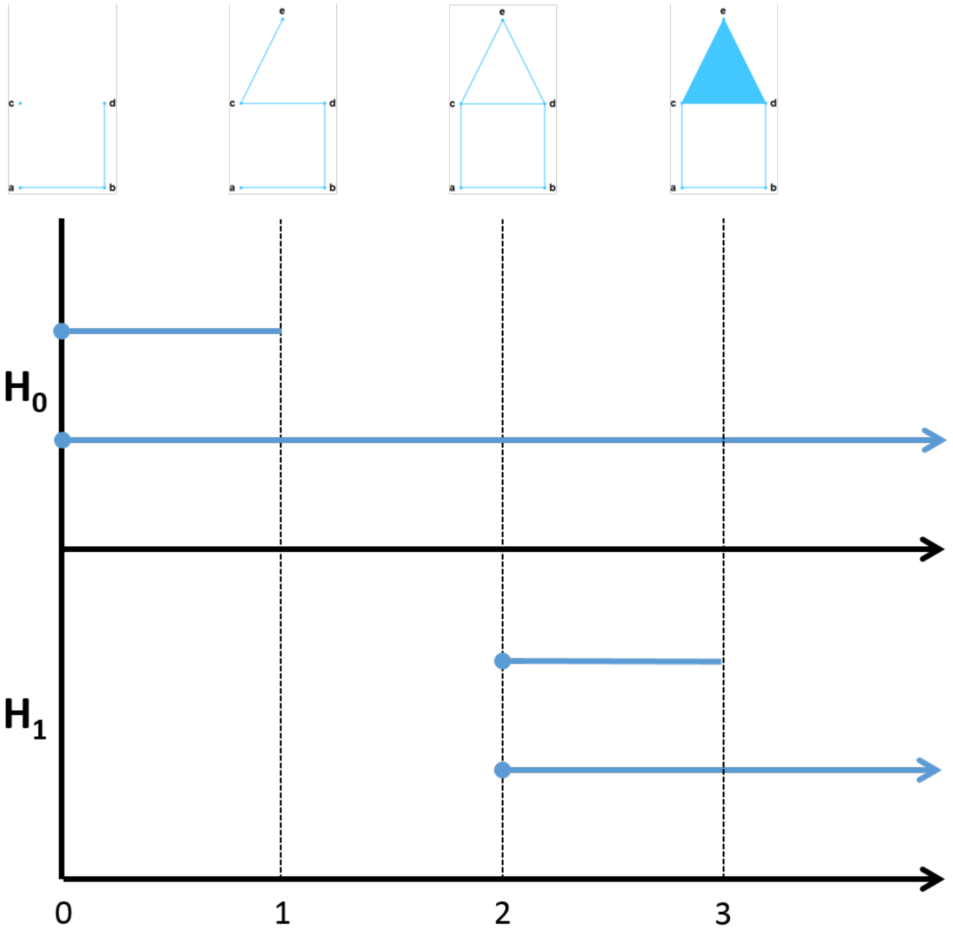

Supplemental Figure S2 2. Barcode representation of persistent homology of K

The 0-dimensional barcodes display the information of the evolution of connected components and in fact, a dendrogram can be constructed out of it. For example, at time zero there are two connected components, the isolated point and the the rest. At time one everything is connected. The barcode $(0,1)$ represents the isolated point which becomes part of the other connected component from time 1 on.

The 1-dimensional barcodes display the information of the evolution of cycles, for example at time 0 and time 1 there are no cycles, from time 1 there are cycles and at time 3 one of the cycles gets filled. The barcode $(2,3)$ represents a cycle with birth time 2 and death time 3, the triangle on the top appears at time 2 and gets filled at time 3. The barcode $(2,\infty)$ represents the square on the bottom that appears at time 2 and persists over time.

For a collection of data points with a notion of similarity, we calculate the barcodes of the persistence homology of the Rips complex associated to the data, the filtration in this case corresponds to the paramater ε

Fig S2 3 shows an application of persitence homology: we take 60 random points on a rotated ellipse, calculate the persistence homology in dimension 0 (black barcodes) and dimension 1 (red barcode) and we reconstruct the cycle detected on the 1-dimensional barcode (red). Note that the cycle misses some points.

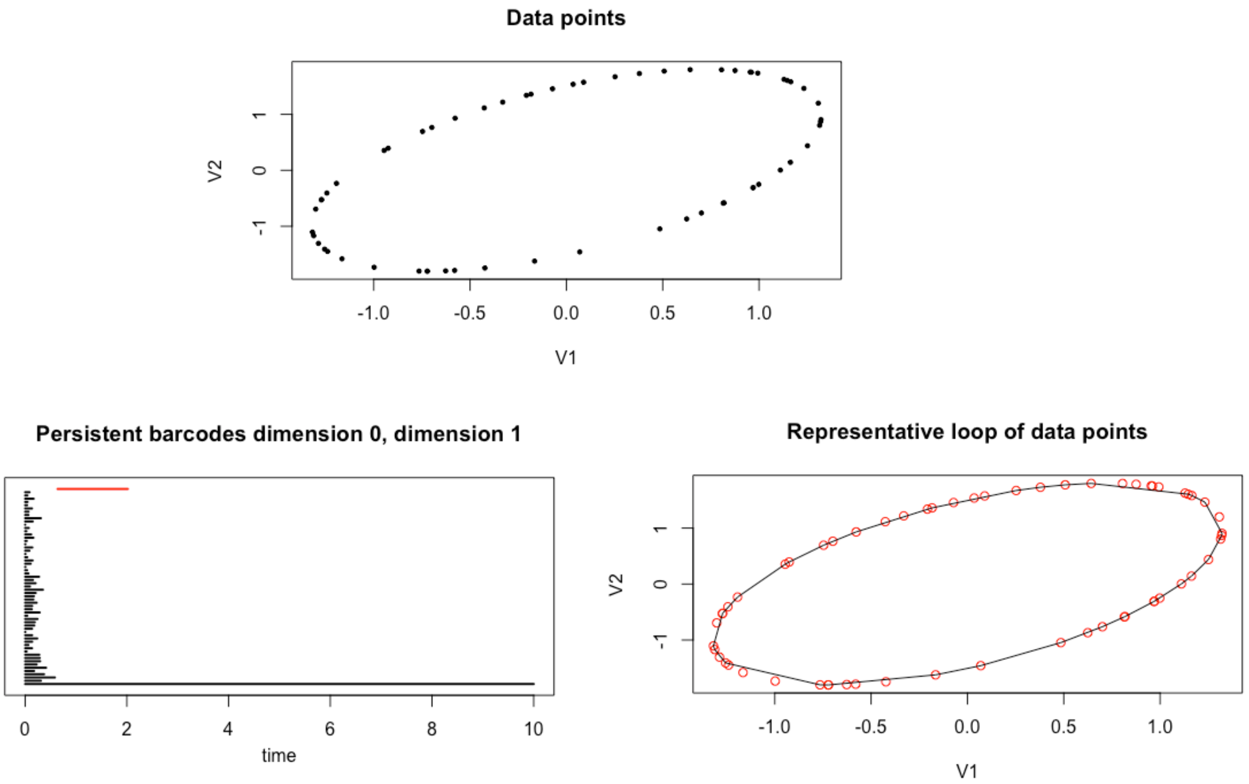

Supplemental Figure S2 3. Data points on a rotated ellipse (up), barcode representation of persistent homology of random points on an ellipse (left) and reconstruction of the cycle on an ellipse (right).

Mapper

To obtain a useful representation of our data we visualize it as its associated graph.

From the filter function $f : X \to \mathbb{R}$ we consider the cover of connected components of the preimages of the cover of $\mathbb{R}$ by overlapping intervals In. The mapper algorithm calculates the Čech complex of this cover. We visualize the 1-skeleton of the mapper complex, i.e. the undirected graph that has vertices in the connected components of the preimages $f^{-1}(I_n)$ and edges if two components intersect.

The input of the method *Mapper* is a collection of points with a distance function and a filter, a function defined on the collection of points. The filter is used to define pieces that cover the collection of points. We apply a clustering algorithm to each piece to obtain a set of local clusters. These are the vertices of the graph. Edges are added to the graph in the following way: two local clusters are connected if they have points in common.

The implementation of mapper1D subdivides the range of the filter function into intervals with a fixed percentage of overlap. The size of each interval and its shifting in order to have a precise fixed amount of overlap is calculated like this:

$$Interval_{size} = \frac{Max_{Filter} - min_{Filter}}{Num_{Intervas}(1 - Overlap) + Overlap)}$$

$$Step_{Size} = Interval_{size} * (1 - Overlap)$$

The maximum possible dimension of the mapper complex can be estimated as follows:

For a $(k+1)$-dimensional face to exist

$$kStep_{size} < Interval_{size}$$

therefore

$$k < \frac{1}{1 - Overlap}$$

So for $0 \leq Overlap \leq \frac{1}{2}$ we have only edges, for $\frac{1}{2} \leq Overlap \leq \frac{2}{3}$ we have 2-dimensional faces.

Any clustering algorithm can be used, in this paper we use agglomerative hierarchical clustering. Figure S2 4 provides a visual road map through the *Mapper* algorithm applied to a sampled set.

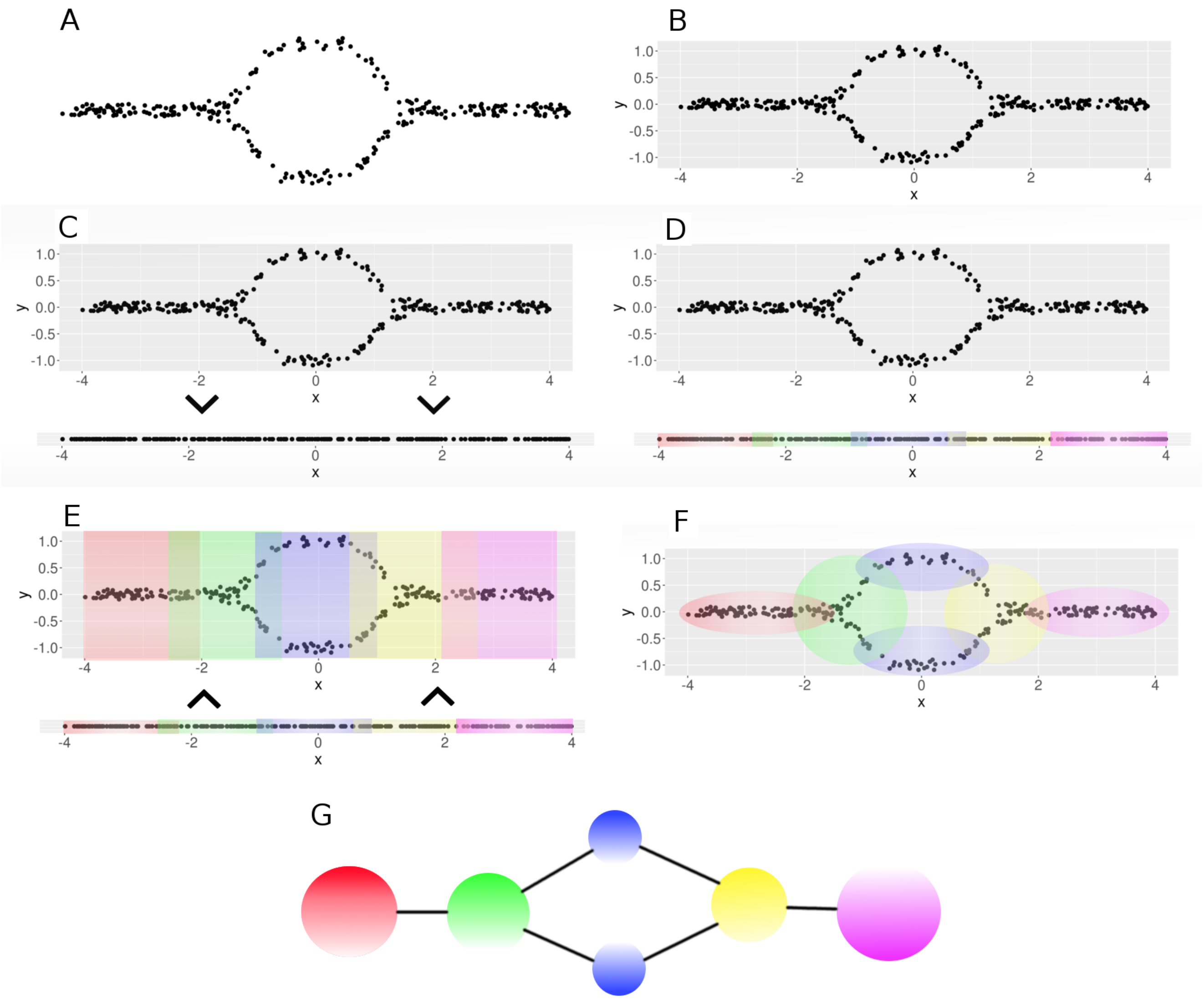

Supplemental Figure S2 4. A given data set is used at the beginning (image A), for this example the points form a straight line with a circle opening in the middle. For convenience we will use the euclidean distance to calculate the distance between each pair of points, so we visualise the points in a metric grid (image B). We then project onto the x coordinate and regard this as our filter function (image C). Next, we divide the filtered values into overlapping intervals of the same length (image D) and construct their pre-images over the original set (image E). Notice how each pair of overlapping intervals is responsible for two different intersecting subsets of points in the original set. Now, inside each pre-image, we run a clustering scheme to detect natural clusters (connected components) among the subset of points (Image F). Each cluster is now regarded as a node in a graph and will have edges between them if their intersection is non empty (Image G).

1.3 Persistent homology of the genetic distance of malaria cases from Guapi region

We applied persistent homology algorithm to the genetic data from Guapi (total data: 98 cases). For population A (20 cases), population B (42 cases), and population C (36 cases). Distance does not take into account time.

Note that when we consider all cases (populations A,B,C), figure S2 5, there are cycles (red barcodes), but when we take each group (all times) separately the are no cycles, fig S2 6.

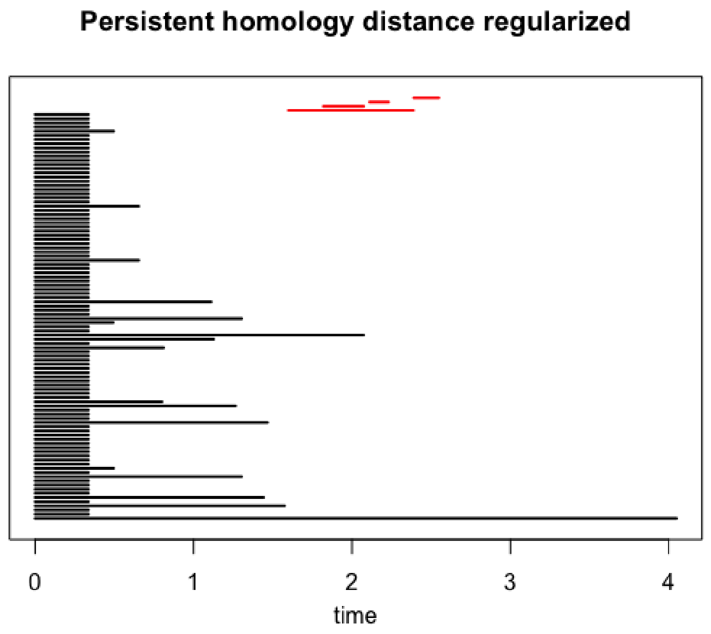

Supplemental Figure S2 5. Barcode representation of persistent homology of all genetic data in Guapi

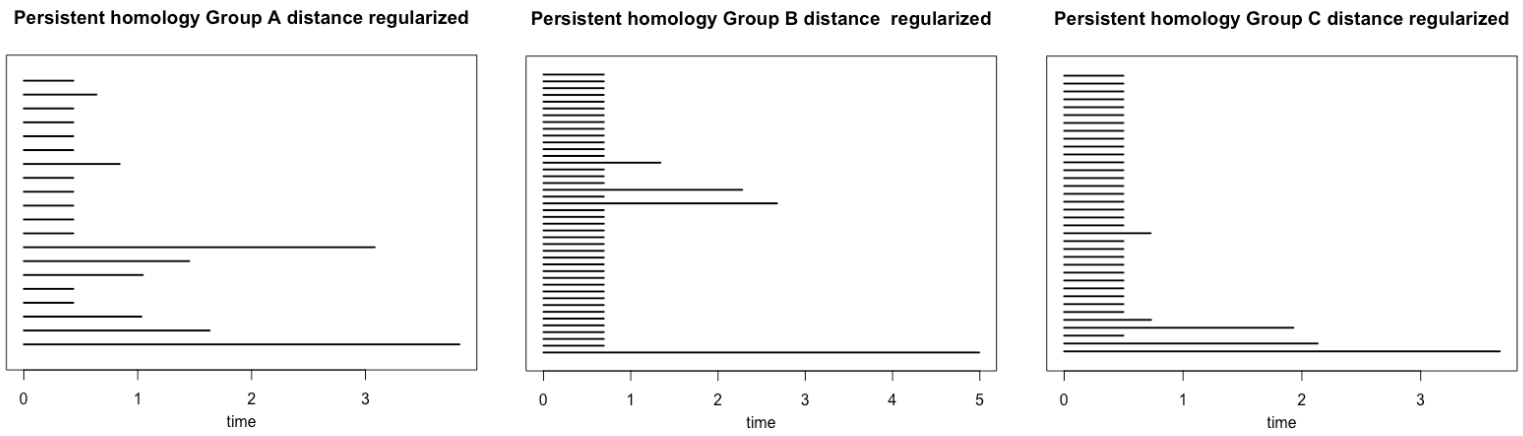

Supplemental Figure S2 6. Barcode representation of persistent homology of by groups in Guapi.

We explored the time dependence of the existence of cycles, for this we first took the same overlapping intervals used in the mapper algorithm and cases from all groups (A,B,C).

Interval	Starting day	Ending day
1	0.0000	248.4337
2	86.95181	335.38554
3	173.9036	422.3373
4	260.8554	509.2892
5	347.8072	596.2410
6	434.7590	683.1928
7	521.7108	770.1446
8	608.6627	857.0964
9	695.6145	944.0482
10	782.5663	1031

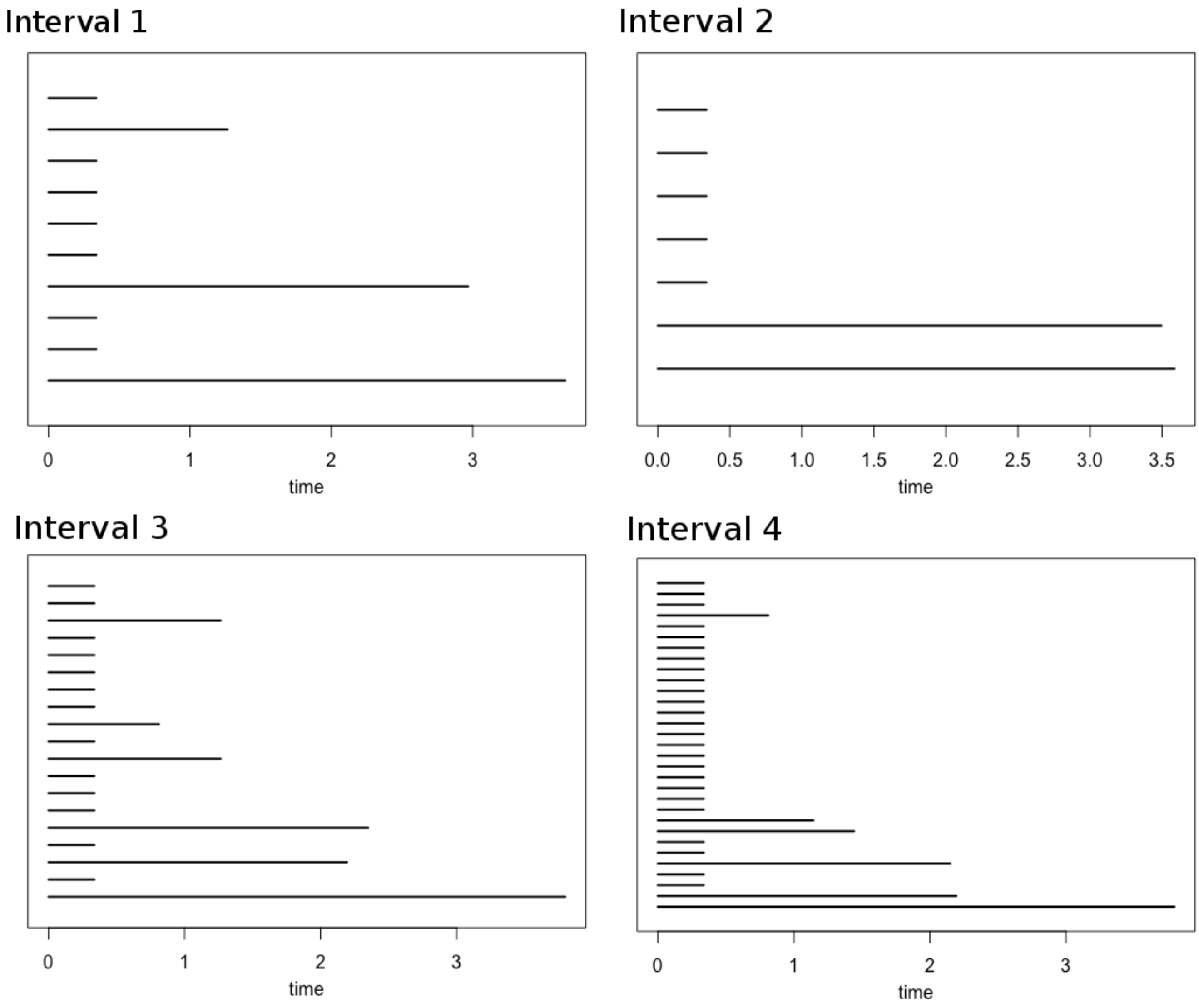

Supplemental Figure S2 7. Barcode representation of persistent homology of cases in intervals 1,2,3 and 4.

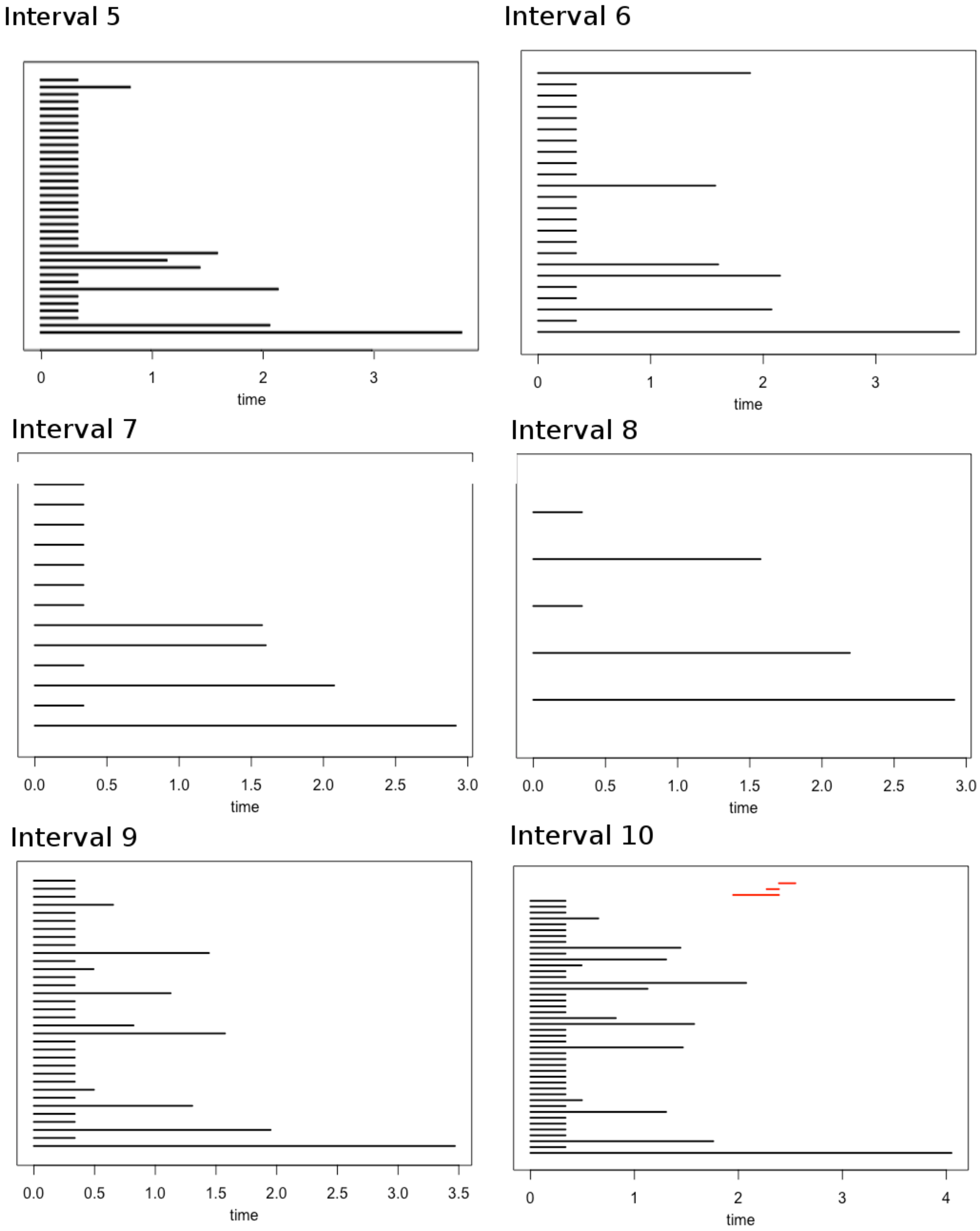

Supplemental Figure S2 8. Barcode representation of persistent homology of cases in intervals 5,6,7,8, 9 and 10.

Note that only in the last interval we observe three cycles (barcodes of dimension 1).

From the barcodes of the total cases (Fig S2 5), we expect to see 4 cycles and by looking interval by interval we just observe three cycles in the last interval. If enlarging the intervals (precisely taking the union of two consecutive ones) we find another cycle in the union of the intervals 6-7, three cycles in the union of the intervals 9-10 and none in the other unions of consecutive intervals (not shown).

Interval	Starting day	Ending day
1 and 2	0.0000	335.3855
2 and 3	86.95181	422.33735
3 and 4	173.9036	509.2892
4 and 5	260.8554	596.2410
5 and 6	347.8072	683.1928
6 and 7	434.7590	770.1446
7 and 8	521.7108	857.0964
8 and 9	608.6627	944.0482
9 and 10	695.6145	1031

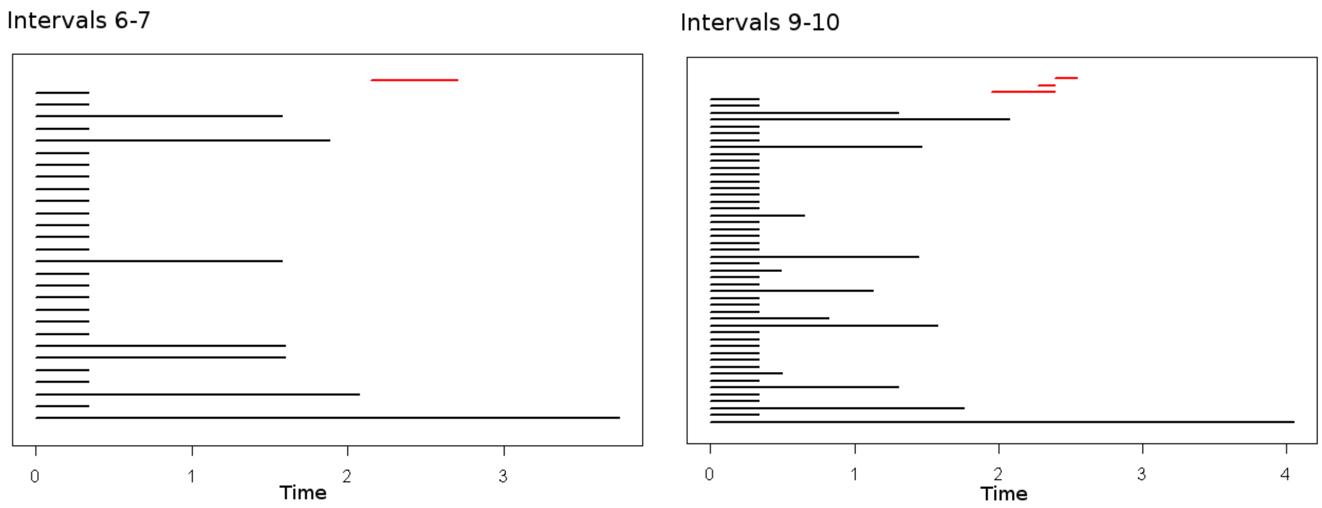

Supplemental Figure S2 9. Barcode representation of persistent homology of cases in interval 6-7 and 9-10

Interestingly, cycles only appear during peaks, suggesting the presence of recombinants during extended epidemics.

2 Social Network Analysis (SNA) of the results of Topological Data Analysis (TDA)

The *Mapper* implementation results in a graph connecting clusters. The aim of this paper, was to combine genetic and epidemiologic variables with TDA. However, the output of such analysis is difficult to interpret and often counter intuitive. To appropriately study the questions we intended to address, we further developed the analysis incorporating SNA of the TDA results. Simple descriptive network statistics were calculated to characterise the relative importance of cases and locations across time and space.

2.1 Aims

The purpose of this study was to describe the role of heterogeneity of infection upon parasite genetic diversity from a micro-epidemiological perspective. This was done over spatial and temporal scales where most of the epidemic and genetic dynamics could be observed. TDA analysis, combined with SNA, enabled us to:

- Understand how parasite subpopulations are connected over space; this is, determining whether subpopulations remain constrained to specific locations over time, or if they vary over spatial and temporal dimensions, and if patterns existed. TDA and SNA enabled us to understand local dynamics in terms of sources and sinks of parasite populations.

- Describe the role of heterogeneity of infection on epidemic and inter-epidemic time-periods over time and space; this is, studying genetic relatedness of single parasite cases over space and time, across heterogeneous epidemic characteristics.

- Characterise geographical regions in terms of their epidemiological relevance, including both genetic and epidemiological characteristics; this is, understanding the aggregated effect of heterogeneous geographic locations on the dynamics of parasite subpopulations and disease burden on segregated human populations.

2.1.1 Hypotheses

We hypothesised that observable patterns exist and deviate from the null hypothesis of random connections across space and time (the resulting networks will diverge from random or probabilistic networks). Since one of the biggest computational challenges is to estimate the range of possible networks of a given graph due to its complexity (2^n), we focused on descriptive statistics of two sub-graphs (cases, locations).

Our hypotheses were as follows:

- The single case parasite network will be characterized by epidemic and inter-epidemic time-periods. Cases contained in epidemic clusters will be highlighted by the largest Pagerank values (since all the cases in an epidemic cluster will point towards the node which connects the cluster to another cluster), whereas cases between epidemics will be characterized by large betweenness centrality values (cases which connect large epidemic clusters which are not interconnected). Pagerank and betweenness centrality can, but need not be proportional for each case (cases can be high in one, both, or none).

- Case centrality (both pagerank and betweenness) will highlight epidemic effects on genetic diversity of subpopulations, such as clone amplifications due to an epidemic, or bottlenecks due to random sampling after epidemic events.

- Geographic networks will describe different segregated and interconnected human populations. Each of these populations will be connected to others by the degree of genetic diversity and epidemic characteristics. As such, locations and human populations can be characterized as sources (high out-degree) or sinks (high in-degree), but most importantly, the location network will characterize human mobility as described by epidemic and genetic dynamics. Thus, locations with high betweenness centrality will describe nodes that connect populations which are otherwise isolated, such as transportation hubs. Locations with high out-degree centrality are likely the result of epidemic outbreaks, pointing to locations where parasite cases with similar genetic characteristics are observed, either preceding in time or in future periods.

2.2 Network Centrality Measures

Centrality measures in networks describe the relative importance of nodes of the graph under a certain criteria. The following definitions of centrality measures describe the variables used in our study.

2.3 Betweenness Centrality

Betweenness centrality is a measure based on the shortest path (i.e the path that uses the least amount of edges for unweighted graphs or the path with the smallest total weight of its edges for weighted graphs) between two specific nodes. This measure of centrality was formalised by LC Freeman in 1977[60] and can be defined like this:

Definition 11 *For a given graph G, the betweenness centrality $b(v)$ of a node $v \in G$ is:*

$$b(v) = \sum_{a \neq b \neq v} \frac{SP_{ab}(v)}{SP_{ab}}$$

where SP_{ab} is the total amount of shortest paths from a to b and $SP_{ab}(v)$ the total amount of shortest paths from a to b that pass through v.

Intuitively, betweenness centrality detects "hubs" in the structure of the network, nodes that if removed will make it harder or impossible to travel across the graph.

2.4 Pagerank Centrality

Pagerank Centrality was first proposed in 1997 by Larry Page and Sergey Brin[61] and is designed to identify the most relevant node in a directed network if one interprets the edges as a direct reference. A formal definition is as follows:

Definition 12 *Given an adjacency matrix A of a graph G, the Pagerank centrality $pr(v_i)$ of a node $v_i \in G$ is the value that satisfies the equation:*

$$pr(v_i) = \alpha \sum_j A_{ji} \frac{v_j}{L_j} + \frac{1-\alpha}{|G|}$$

Where $\alpha \in [0,1]$ *and*

$$L_j = \sum_i A_{ji}$$

This centrality is a variation of the eigenvector centrality, where the value of a node is extracted from the vector x such that: $Ax = \lambda x$ (for some $\lambda \in \mathbb{R}$). If one regards the edges as direct references, elements with high Pagerank values represent nodes referenced by other high ranking nodes.

2.5 Results

Two networks with heterogeneity of degree distributions resulted from combining SNA and TDA. Figures S2 10 and S2 11 show variation of betweenness and Pagerank centrality among cases. Note that cases with highest Pagerank and betweenness centrality were observed during epidemic years (2015, 2017) while cases with high betweenness centrality happened in inter-epidemic years (2016). Interestingly, a selection of cases of subpopulation B had higher centrality values (Pagerank and betweenness). A selection of cases of subpopulation C were also observed with high centrality values. Subpopulation A was observed to have lower maximum centrality values than those cases for the other subpopulations. Tables S2 1 and S2 2 show the most central cases for Pagerank and betweenness respectively.

Similarly, by contracting edges in the point intersection network, a contracted geographical graph (Geographic Network) was constructed. By merging adjacent nodes whose cases had the same geographical location, a network whose nodes represent a specific time, location and genetic diversity was built. Note that this is different than simply merging nodes by location, which would change the topological properties of the network with respect to the 1-skeleton. The centrality values for this nodes are shown in Figures S2 10 and S2 11. Three nodes were observed with high centrality values for both Pagerank and betweenness: *Bagrero-2*, *Carmelo-1* and *Guapi-1*. *Bagrero-2* was observed with high centrality values at both epidemic (2015) and inter-epidemic (2016) years, *Carmelo-1* at both inter-epidemic (2016) and epidemic periods (2017), while *Guapi-1* only during epidemic interval (2017). Only one node was observed with high betweenness and low Pagerank: *Cuerval-1*, included in the interval ranging from Apr-2016 to Nov-2016 (inter-epidemic).

Results of subpopulation geographical distribution are discussed in detail in the main text.

Sample ID	Group	Origin	Date (mm/dd/yy)	Pagerank Cent.	Betweenness Cent.
GU-397	C	Guapi	6/16/17	0.18	1360
GU-129	B	Guapi	11/24/15	0.15	2891
GU-374	B	Carmelo	5/7/17	0.08	2584
GU-78	C	Guapi	4/20/15	0.03	556
CU-11	C	Cuerval	2/2/16	0.03	165.9
GU-118	A	Carmelo	3/1/16	0.03	75.5
GU-109	B	El Firme	2/19/16	0.03	208.5
CU-25	C	Cuerval	7/1/16	0.01	4
GU-124	A	Guare	9/14/15	0.01	190

Supplemental Table S2 1. Top ten cases with highest Pagerank centrality in the case (Point Intersection) network

Sample ID	Group	Origin	Date (mm/dd/yy)	Pagerank Cent.	Betweenness Cent.
GU-129	B	Guapi	11/24/15	0.15	2891
GU-374	B	Carmelo	5/7/17	0.08	2584
CU-12	B	Cuerval	4/24/16	0.01	2323.5
GU-397	C	Guapi	6/16/17	0.18	1360
CU-31	B	Cuerval	11/15/16	0.01	1127
CU-30	B	Cuerval	11/7/16	0.00	1127
GU-78	C	Guapi	4/20/15	0.03	556
GU-109	B	El Firme	2/19/16	0.03	208.5
CU-6	C	Playa Chacon	12/3/15	0.00	203

Supplemental Table S2 2. Top ten cases with highest betweenness centrality in the case (Point Intersection) network

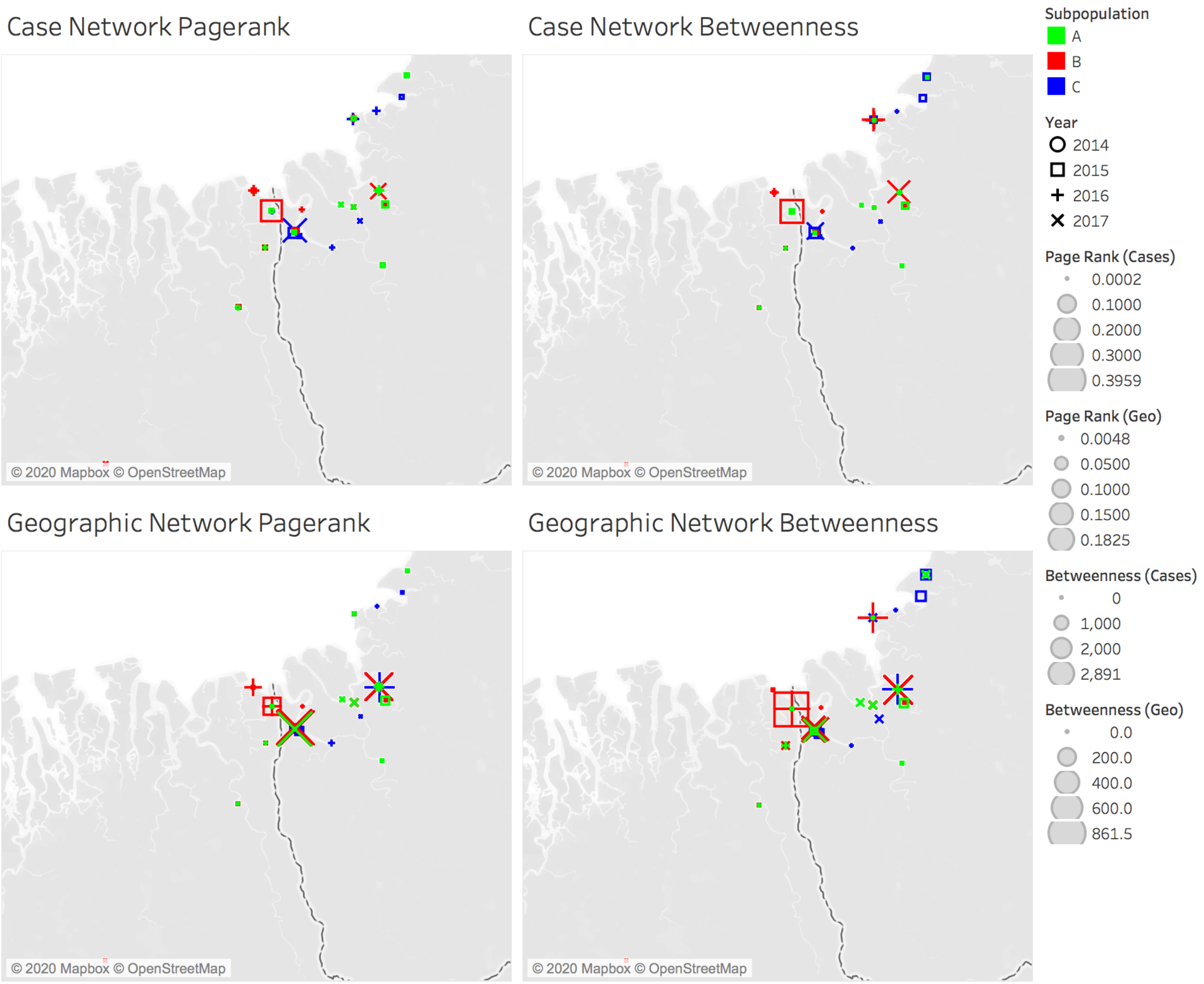

Supplemental Figure S2 10. Pagerank and betweenness centrality for the case and geographic networks. Maps created using Tableau 2019.4 (https://www.tableau.com/). Source: OpenStreetMap[62], © OpenStreetMap contributors, under an Attribution-Share-Alike 2.0 Generic licence (https://creativecommons.org/licenses/by-sa/2.0/).

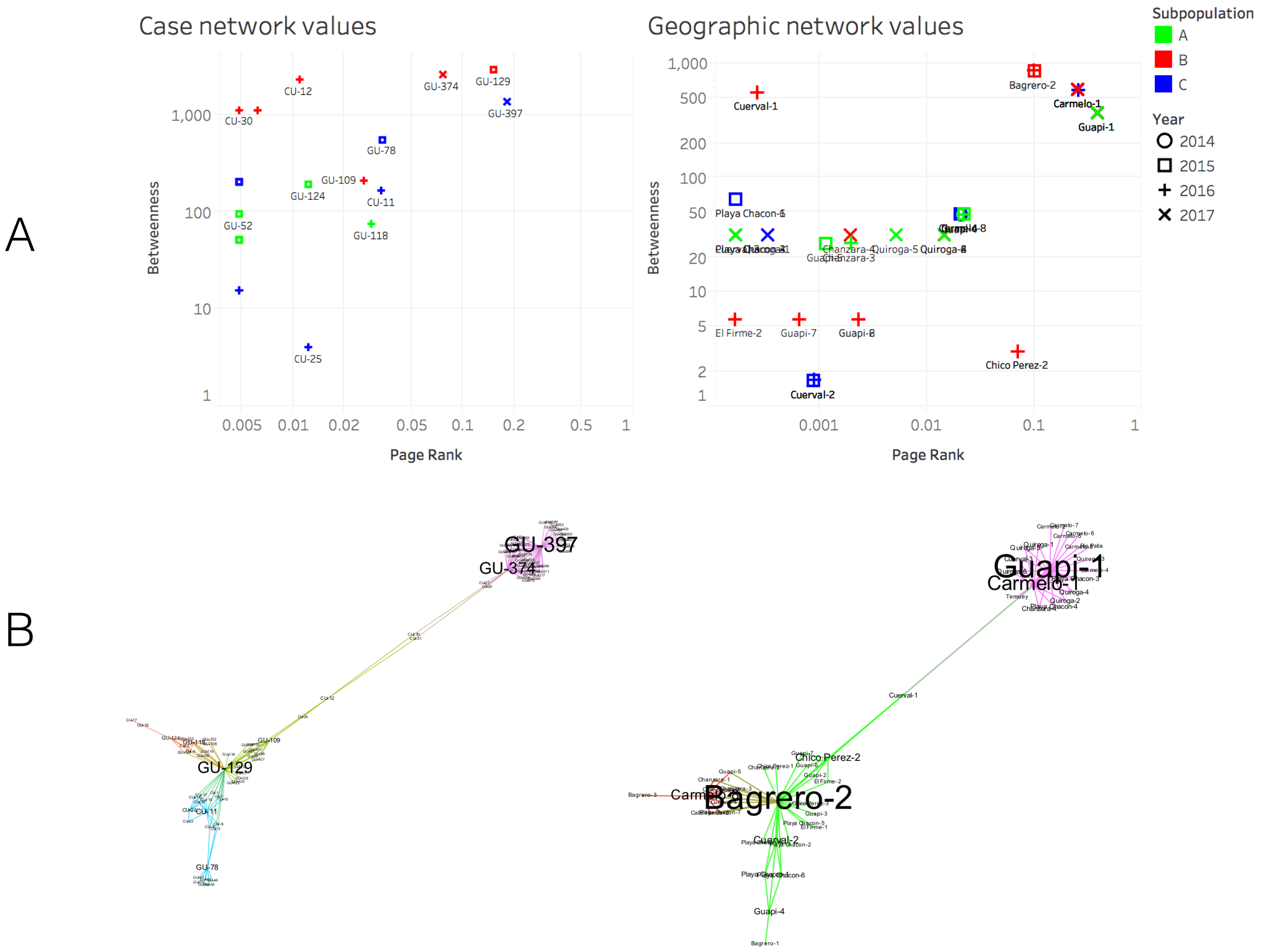

Supplemental Figure S2 11. A. Pagerank and betweenness centrality values for the different nodes in the case and contracted geographic networks. B. Case (left) and geographic (right) networks.

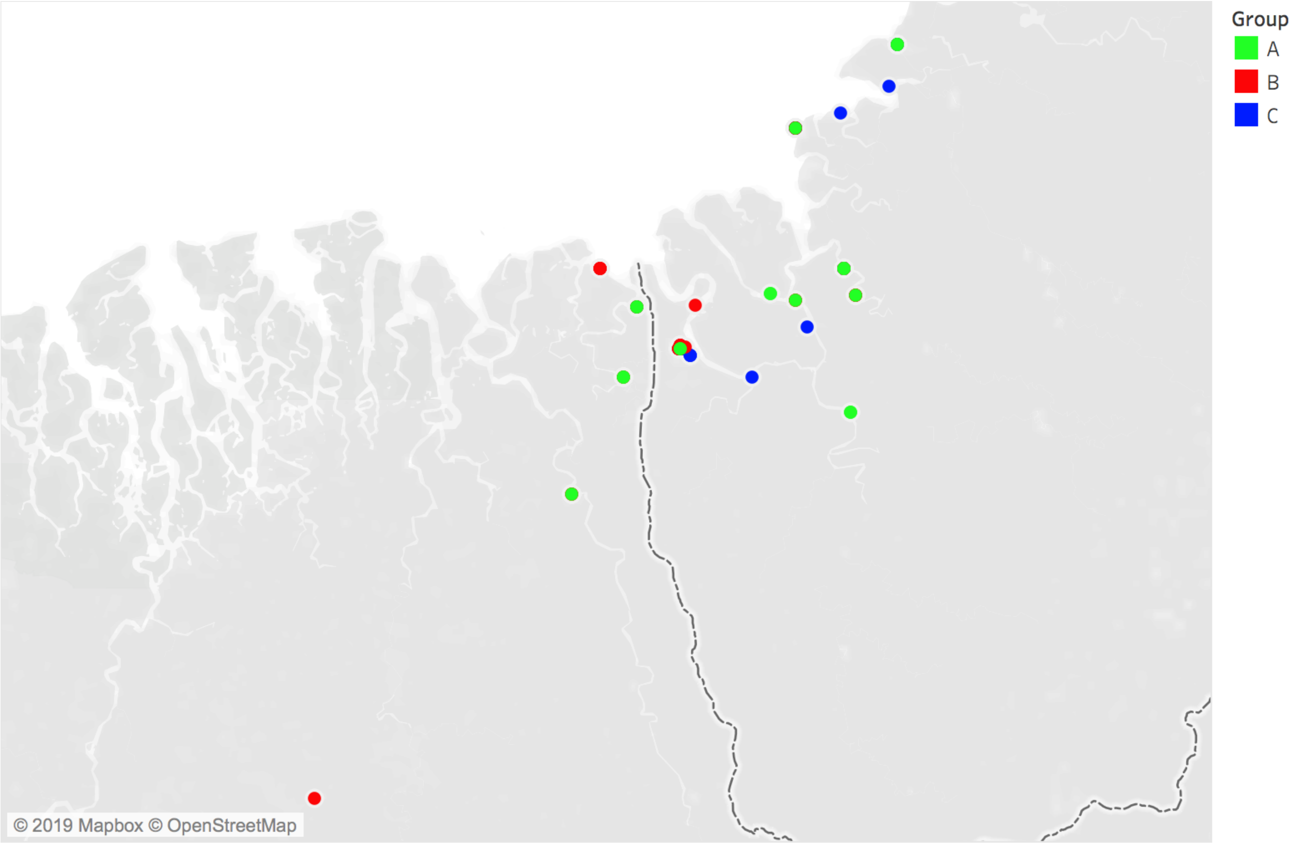

Supplemental Figure S2 12. Geographical location of the different clonal groups. Clonal groups correspond to the longest zero cost paths in the graph constructed using the pairwise genetic distances. This means that for any two samples a and b in the same clone group G, there are samples $v_1, \ldots v_n \in G$ such that: $a = v_1$, $b = v_n$ and the pairwise distance between v_i and v_{i+1} is zero. Map created using Tableau 2019.4 (https://www.tableau.com/). Source: OpenStreetMap[62], © OpenStreetMap contributors, under an Attribution-Share-Alike 2.0 Generic licence (https://creativecommons.org/licenses/by-sa/2.0/).

References

1. Singh G, Memoli F, Carlsson G. Topological Methods for the Analysis of High Dimensional Data Sets and 3D Object Recognition. Prague, Czech Republic: Eurographics Association; 2007. p. 91–100.
2. Lum PY, Singh G, Lehman A, Ishkanov T, Vejdemo-Johansson M, Alagappan M, et al. Extracting insights from the shape of complex data using topology. 2013;3:1236 EP –.
3. Carlsson G. Topology and data. Bulletin of the American Mathematical Society. 2009;46:255–308.
4. Carlsson G. Topological pattern recognition for point cloud data. Acta Numerica. 2014;23:289–368.
5. Chan JM, Carlsson G, Rabadan R (2013) Topology of viral evolution. Proc Natl Acad Sci USA 110:18566-18571
6. Cámara PG, Levine AJ, Rabadán R (2016) Inference of ancestral recombination graphs through topological data analysis. PLoS Comput Biol 12:Article ID e1005071
7. Emmett K, Rosenbloom D, Camara P, Rabadan R (2014) Parametric inference using persistence diagrams: a case study in population genetics. arXiv:1406.4582
8. Camara PG, Rosenbloom DI, Emmett KJ, Levine AJ, Rabadan R. Topological Data Analysis Generates High-Resolution, Genome-wide Maps of Human Recombination. Cell Syst. 2016;3:83–94.
9. Emmett K.J., Rabadan R. (2014) Characterizing Scales of Genetic Recombination and Antibiotic Resistance in Pathogenic Bacteria Using Topological Data Analysis. In: Slezak D., Tan AH., Peters J.F., Schwabe L. (eds) Brain Informatics and Health. BIH 2014. Lecture Notes in Computer Science, vol 8609. Springer, Cham

10. Cámara, Pablo G. Topological methods for genomics: present and future directions Current opinion in systems biology vol. 1 (2016): 95-101.

11. Emmett K, Schweinhart B, Rabadán R (2016) Multiscale topology of chromatin folding. In: Proceedings of the 9th EAI international conference on bio-inspired information and communications technologies (formerly BIONETICS), BICT'15. ICST (Institute for Computer Sciences, Social-Informatics and Telecommunications Engineering), Brussels, pp 177-180

12. Rizvi A, Camara P, Kandror E, Roberts T, Schieren I, Maniatis T, Rabadan R (2017) Single-cell topological RNA-seq analysis reveals insights into cellular differentiation and development. Nat Biotechnol 35:551-560. doi:10.1038/nbt.3854

13. Ibekwe AM, Ma J, Crowley DE, Yang CH, Johnson AM, Petrossian TC, et al. Topological data analysis of Escherichia coli O157:H7 and non-O157 survival in soils. Frontiers in Cellular and Infection Microbiology. 2014;4:122.

14. Nielson JL, Paquette J, Liu AW, Guandique CF, Tovar CA, Inoue T, et al. Topological data analysis for discovery in preclinical spinal cord injury and traumatic brain injury. Nature Communications. 2015;6:8581.

15. Hinks T.S., Zhou X., Staples K.J., Dimitrov B.D., Manta A., Petrossian T. Innate and adaptive T cells in asthmatic patients: relationship to severity and disease mechanisms. J Allergy Clin Immunol. 2015;136:323–333

16. Hinks TS, Brown T, Lau LC, Rupani H, Barber C, Elliott S, Ward JA, Ono J, Ohta S, Izuhara K, et al. Multidimensional endotyping in patients with severe asthma reveals inflammatory heterogeneity in matrix metalloproteinases and chitinase 3-like protein 1. J Allergy Clin Immunol. 2016

17. Li, Li et al. "Identification of type 2 diabetes subgroups through topological analysis of patient similarity" Science translational medicine vol. 7,311 (2015): 311ra174.

18. Torres, Brenda Y et al. "Tracking Resilience to Infections by Mapping Disease Space" PLoS biology vol. 14,4 e1002436. 18 Apr. 2016, doi:10.1371/journal.pbio.1002436

19. G. Carlsson, T. Ishkhanov, V. De Silva, and A. Zomorodian. *On the local behavior of spaces of natural images*. International journal of computer vision, 76(1):1–12, 2008.

20. Perea JA, Deckard A, Haase SB, Harer J (2015) Sw1pers: sliding windows and 1-persistence scoring; discovering periodicity in gene expression time series data. BMC Bioinform 16:Article ID 257

21. Sardiu ME, Gilmore JM, Groppe B, Florens L, Washburn MP. Identification of Topological Network Modules in Perturbed Protein Interaction Networks. Scientific Reports. 2017;7:43845.

22. Kovacev-Nikolic V, Bubenik P, Nikolic D, Heo G (2014) Using persistent homology and dynamical distances to analyze protein binding. arXiv:1412.1394.

23. Gameiro M, Hiraoka Y, Izumi S, Kramár M, Mischaikow K, Nanda V (2015) A topological measurement of protein compressibility. Jpn J Ind Appl Math 32:1-17

24. Xia K, Wei G-W (2014) Persistent homology analysis of protein structure, flexibility, and folding. Int J Numer Methods Biomed Eng 30:814-844

25. Xia K, Li Z, Mu L (2016) Multiscale persistent functions for biomolecular structure characterization. arXiv:1612.08311

26. Bhattacharya S, Ghrist R, Kumar V (2015) Persistent homology for path planning in uncertain environments. IEEE Trans Robot 31:578-590

27. Pokorny FT, Hawasly M, Ramamoorthy S (2016) Topological trajectory classification with filtrations of simplicial complexes and persistent homology. Int J Robot Res 35:204-223

28. Vasudevan R, Ames A, Bajcsy R (2013) Persistent homology for automatic determination of human-data based cost of bipedal walking. Nonlinear Anal Hybrid Syst 7:101-115

29. Chung MK, Bubenik P, Kim PT (2009) Persistence diagrams of cortical surface data. In: Prince JL, Pham DL, Myers KJ (eds) Information processing in medical imaging. Lecture notes in computer science, vol 5636. Springer, Berlin, pp 386-397

30. Guillemard M, Boche H, Kutyniok G, Philipp F (2013) Signal analysis with frame theory and persistent homology. In: 10th international conference on sampling theory and applications, pp 309-312

31. Nicolau M, Levine AJ, Carlsson G (2011) Topology based data analysis identifies a subgroup of breast cancers with a unique mutational profile and excellent survival. Proc Natl Acad Sci USA 108:7265-7270

32. DeWoskin D, Climent J, Cruz-White I, Vazquez M, Park C, Arsuaga J (2010) Applications of computational homology to the analysis of treatment response in breast cancer patients. Topol Appl 157:157-164

33. Crawford L, Monod A, Chen AX, Mukherjee S, Rabadán R (2016) Topological summaries of tumor images improve prediction of disease free survival in glioblastoma multiforme. arXiv:1611.06818

34. Singh N, Couture HD, Marron JS, Perou C, Niethammer M (2014) Topological descriptors of histology images. In: Wu G, Zhang D, Zhou L (eds) Machine learning in medical imaging. Lecture notes in computer science, vol 8679. Springer, Cham, pp 231-239

35. Belchi, Francisco and Pirashvili, Mariam and Conway, Joy and Bennett, Michael and Djukanovic, Ratko and Brodzki, Jacek, Lung Topology Characteristics in patients with Chronic Obstructive Pulmonary Disease, Scientific Reports, 1, 5341, ,8,2018

36. Carlsson G, Ishkhanov T, de Silva V, Zomorodian A (2008) On the local behavior of spaces of natural images. Int J Comput Vis 76:1-12

37. Taylor D, Klimm F, Harrington HA, Kramár M, Mischaikow K, Porter MA, Mucha PJ (2015) Topological data analysis of contagion maps for examining spreading processes on networks. Nat Commun 6:Article ID 7723

38. Lo D, Park B (2016) Modeling the spread of the Zika virus using topological data analysis. arXiv:1612.03554

39. Kramár M, Goullet A, Kondic L, Mischaikow K (2013) Persistence of force networks in compressed granular media. Phys Rev E 87:Article ID 042207

40. Kramár M, Goullet A, Kondic L, Mischaikow K (2014) Quantifying force networks in particulate systems. Physica D 283:37-55

41. Hiraoka Y, Nakamura T, Hirata A, Escolar E, Matsue K, Nishiura Y (2016) Hierarchical structures of amorphous solids characterized by persistent homology. Proc Natl Acad Sci USA 113:7035-7040

42. Lee Y, Barthel SD, Dłotko P, Mohamad Moosavi S, Hess K, Smit B (2017) Pore-geometry recognition: on the importance of quantifying similarity in nanoporous materials. arXiv:1701.06953

43. Leibon G, Pauls S, Rockmore D, Savell R (2008) Topological structures in the equities market network. Proc Natl Acad Sci USA 105:20589-20594

44. Gidea M (2017) Topology data analysis of critical transitions in financial networks. arXiv:1701.06081

45. Giusti C, Ghrist R, Bassett D (2016) Two's company and three (or more) is a simplex. J Comput Neurosci 41:1-14

46. Curto C (2017) What can topology tell us about the neural code? Bull, New Ser, Am Math Soc 54:63-78

47. Dłotko P, Hess K, Levi R, Nolte M, Reimann M, Scolamiero M, Turner K, Muller E, Markram H (2016) Topological analysis of the connectome of digital reconstructions of neural microcircuits. arXiv:1601.01580

48. Kanari L, Dłotko P, Scolamiero M, Levi R, Shillcock J, Hess K, Markram H (2016) Quantifying topological invariants of neuronal morphologies. arXiv:1603.08432

49. Lord L-D, Expert P, Fernandes HM, Petri G, Van Hartevelt TJ, Vaccarino F, Deco G, Turkheimer F, Kringelbach M (2016) Insights into brain architectures from the homological scaffolds of functional connectivity networks. Front Syst Neurosci 10:Article ID 85

50. Bendich P, Marron JS, Miller E, Pieloch A, Skwerer S (2016) Persistent homology analysis of brain artery trees. Ann Appl Stat 10:198-218

51. Yoo J, Kim EY, Ahn YM, Ye JC (2016) Topological persistence vineyard for dynamic functional brain connectivity during resting and gaming stages. J Neurosci Methods 267:1-13

52. Dabaghian Y, Brandt VL, Frank LM (2014) Reconceiving the hippocampal map as a topological template. eLife 3:Article ID e03476

53. Sizemore A, Giusti C, Bassett D (2017) Classification of weighted networks through mesoscale homological features. J Complex Netw 5:245-273

54. Pal S, Moore TJ, Ramanathan R, Swami A (2017) Comparative topological signatures of growing collaboration networks. In: Complex networks VIII. Springer, Cham, pp 201-209

55. Carstens CJ, Horadam KJ (2013) Persistent homology of collaboration networks. Math Probl Eng 2013:Article ID 815035

56. Bajardi P, Delfino M, Panisson A, Petri G, Tizzoni M (2015) Unveiling patterns of international communities in a global city using mobile phone data. EPJ Data Sci 4:Article ID 3

57. Topaz CM, Ziegelmeier L, Halverson T (2015) Topological data analysis of biological aggregation models. PLoS ONE 10:Article ID e0126383

58. Maletic S, Zhao Y, Rajkovic M (2015) Persistent topological features of dynamical systems. arXiv:1510.06933

59. Zhu X (2013) Persistent homology: an introduction and a new text representation for natural language processing. In: Proceedings of the twenty-third international joint conference on artificial intelligence, IJCAI '13, Beijing, China AAAI Press, Menlo Park, pp 1953-1959

60. Freeman, L. C. A set of measures of centrality based on betweenness. Sociometry 35–41 (1977).

61. Page, L., Brin, S., Motwani, R. & Winograd, T. Pagerank: Bringing order to the web. Tech. Rep., Stanford Digital Libraries Working Paper (1997).

62. OpenStreetMap contributors. Planet dump retrieved from https://planet.osm.org . https://www.openstreetmap.org, 2017.
